# New Perspectives on European Wildcat ( *Felis silvestris* , Schreber 1777) Habitat Suitability in Britain: Integrating Fossil Records to Improve Baselines for Reintroduction

**DOI:** 10.1002/ece3.72126

**Published:** 2025-09-25

**Authors:** Mollie Mills, Laura Hemmingham, Danielle Schreve

**Affiliations:** ^1^ Department of Geography Royal Holloway University of London Surrey UK; ^2^ School of Geographical Sciences University of Bristol Bristol UK

**Keywords:** conservation palaeobiology, European wildcat, *Felis silvestris*, habitat suitability model, niche truncation, Pleistocene fossils

## Abstract

In Britain, the European wildcat ( *Felis silvestris* , Schreber 1777) is located exclusively in Scotland. Considered to be Critically Endangered, the Scottish population is on the brink of extinction due to continued population decline through anthropogenic activities, contact with domestic and feral cats and habitat loss. Urgent and effective conservation strategies, including breeding and translocation programs, are required to support existing populations and aid the species' recovery. Understanding the species' environmental requirements and identifying suitable areas for wildcat reintroductions is therefore essential. However, using the species' modern range characteristics for habitat suitability projections is problematic due to extensive range contraction, resulting in the wildcat now occupying a reduced set of environmental conditions. Incorporating the wildcat's historic and recent fossil distribution data into such projections can significantly improve understanding of the species–environment relationship, prior to extensive range contraction. Here, we use the wildcat's historic and Holocene fossil record in Britain to construct multi‐temporal habitat suitability models (HSMs) under current climate conditions. The models identified elevation and human impact as important variables for predicting wildcat environmental suitability. Our results show that modern wildcat populations have suffered species‐environment truncation and now occupy only a portion of their potential environmental range across Britain, with a reduced realised niche breadth. The addition of Holocene fossil data into the models increases the amount of excellent and good habitat predicted by 37% and 197%, respectively, extending into other parts of Scotland, as well as England and Wales, and decreases predicted poor suitability conditions by 97.7%, thereby offering new avenues for reintroduction programs. Overall, our study underlines the benefits of using multi‐temporal HSMs to represent species–environment relationships more accurately and to improve habitat suitability projections under current and future climate change scenarios.

## Graphical abstract

This study models habitat suitability for the European wildcat ( *Felis silvestris* , Schreber 1777) in Britain under current climate conditions using modern, historic and Holocene fossil occurrence records and associated (palaeo) environmental data. Our study highlights how models calibrated with multi‐temporal ecological data are able to capture the potential environmental range of a species' more accurately to improve the effectiveness of conservation recovery programs. This is significant for the European wildcat in Britain, a Critically Endangered population that requires urgent conservation action.

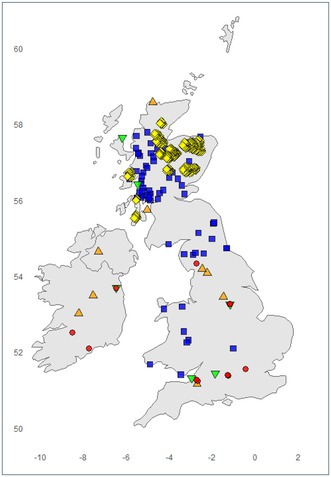

## 1 Introduction

The European wildcat ( *Felis silvestris* , Schreber 1777) is a polytypic species that ranges from Britain to European Russia, excluding Scandinavia (Gerngross et al. 2023). As the last remaining wild felid species in Britain (MacDonald et al. 2010), this animal was once widespread but has undergone extensive range contraction and is now restricted to fragmented populations across northern Scotland (Kilshaw et al. 2016). Although continental European wildcats are considered to be of ‘Least Concern’ by the International Union for Conservation of Nature (IUCN), the Scottish population is on the brink of extinction (Gerngross et al. 2023). Threatened by hybridisation, competition and disease transmission from feral domestic cats (Pierpaoli et al. 2003), as well as habitat loss, prey decline and accidental persecution, current population estimates range between 30 and 430 mature individuals (Kilshaw 2015; Mathews et al. 2018; Breitenmoser et al. 2019). However, population estimates remain uncertain due to limited spatial data on the distribution of wildcats in Scotland and complications in distinguishing genetically pure wildcats from hybrids (Silva, Rosalino, et al. 2013; Kilshaw et al. 2016).

As the most endangered mammalian carnivore in Britain, the wildcat is a conservation priority in Scotland (Kitchener et al. 2005; Breitenmoser et al. 2019; Howard‐McCombe et al. 2021). Scottish conservation organisations are working to reduce the threats faced by wildcats, particularly hybridisation, and to conserve existing populations (Daniels et al. 2001; Kitchener et al. 2005; Senn et al. 2018). Nevertheless, based on 20 pelage characteristics of Scottish wildcat specimens from the last 100 years, only 12% presented the accepted ‘classic’ wildcat pelage characteristics. The remaining 88% were determined to be either hybrids or wild‐living domestic cats (Kitchener et al. 2005). In 2019, a report by the IUCN Cat Specialist Group accordingly determined the current Scottish wildcat population as unviable, stating that recovery and avoiding extinction entirely would only be possible through reintroduction and supplementing reinforcement projects with continental European wildcats (Breitenmoser et al. 2019). The Saving Wildcats Recovery Project has since been established with a focus on breeding genetically pure wildcats for wild release in Scotland (Campbell et al. 2023). The current population of British wildcats is thought to have recolonised from continental Europe via the land bridge approximately 10,000 years ago at the end of the Last Ice Age (Breitenmoser et al. 2019), with all Holocene wildcats from Britain placing within a large west‐central European matrilineage (Mattucci et al. 2015), and the geographical origin of British wildcats being the north‐west coast of Belgium or France (Marr 2016).

Identifying suitable habitat areas for wildcat reintroductions in Scotland and more widely across Britain is therefore crucial for the species' recovery. This may be achieved via the well‐established method of characterising wildcat habitat, based on the perceived optimal environmental suitability of the species' current geographic range (Littlewood et al. 2014). This approach is, however, fraught with problems. Wildcats display a predominant association with mixed or broad‐leaved forests (Sommer and Benecke 2006; Yamaguchi et al. 2015; Klar et al. 2008; Kitchener and O'Connor 2010) and British wildcat populations almost certainly experienced both demographic and range contractions during periods of climatic deterioration in the past, associated with woodland decline (Marr 2016). However, the remaining Scottish population has experienced severe anthropogenic range contraction and may have suffered species‐environment truncation (Faurby and Araújo 2018). Species‐environment truncation occurs when disturbance and regional extirpations cause species to be absent from large portions of their former range, occupying a reduced set of environmental conditions with a restricted realised niche breadth (Scheele et al. 2017; Monsarrat et al. 2019; Sales et al. 2022; Mills et al. 2024). This phenomenon is problematic for conservation practitioners, as contemporary distribution patterns may not reflect full environmental tolerances or habitat preferences for endangered species (Di Marco and Santini 2015; Di Marco et al. 2021; Pang et al. 2022). Indeed, for some species, known as refugees, environmental truncation is so pronounced that they now only occur in suboptimal environmental conditions (Kerley et al. 2012, 2020). Failure to recognise and account for environmental truncation can lead to an underestimation of suitable environmental conditions and restricted or misinformed conservation strategies (Bush et al. 2018; Sales et al. 2022). This is therefore particularly relevant for the last remaining wildcats in Britain.

A method commonly used to reduce the effect of species‐environment truncation is to incorporate historic distribution data into conservation assessments (Grace et al. 2019; Monsarrat et al. 2019). Historic records can provide a longer‐term perspective of species‐environment relationships prior to contemporary range contraction (e.g., Chatterjee et al. 2012; Turvey et al. 2015; Yang et al. 2018; Rodrigues et al. 2019). Despite this, humans have been altering species' ranges for thousands of years; therefore, historic records may themselves still be prone to truncation and bias (Faurby and Svenning 2015). Increasingly, researchers are including fossil records into biodiversity assessments to provide deeper‐time perspectives on species' full environmental relationships and ecological tolerances prior to widespread human disturbance (Dietl and Flessa 2011; Kiessling et al. 2019; Turvey and Saupe 2019; van de Velde et al. 2019). Fossil records have been integrated into biodiversity and conservation assessments, including reintroduction and rewilding projects, for a variety of taxa including birds, corals and mammals (see Lima‐Ribeiro et al. 2017; Archer et al. 2019; Lentini et al. 2018; Jones et al. 2019; Jarvie et al. 2021; Crees et al. 2023; Mills et al. 2024).

As a critically endangered population that has undergone extensive range contraction and which consequently does not occupy its full geographic range today, analysing the wildcat's fossil and historic distribution data in Britain may provide new conservation insights. Historically, the wildcat was widely distributed across Britain. Its disappearance is recorded from southern England by the 16th century, and the species showed a steady decline during the 18th and 19th centuries, until it was entirely absent from Wales and England by the late 1870s (Langley and Yalden 1977; Easterbee et al. 1991). Persecution as vermin and the rise of the sporting estate (requiring protection of game birds from predators), as well as hunting, are considered to be the main drivers for the historic decline (Yalden 1999; Breitenmoser et al. 2019). Wildcat fossils are consistently found in Quaternary deposits across Britain and Ireland, indicating that the species had a much larger spatial distribution and environmental range than the current Scottish population (Kitchener and O'Connor 2010; Yalden 1999). However, the wildcat's fossil record in Britain has not yet been applied to modern conservation research. Its incorporation into habitat suitability projections may not only improve understanding of the species' broader environmental range but also help to identify new areas for reintroduction projects outside Scotland. Restoring the wildcat population across Britain is important not only for recovering biodiversity but also lost ecosystem functions such as controlling wild small mammal populations (Corbett 1979; Silva, Kilshaw, et al. 2013). Wildcats, as meso‐predators, are effective regulators of prey species populations, including various small rodents and the European rabbit ( *Oryctolagus cuniculus* , Linnaeus 1758) which is an invasive species in Britain (Martín‐Díaz et al. 2018; Tossens et al. 2024).

In this study, we model habitat suitability for the European wildcat in Britain under current climate conditions using modern, historic and Holocene fossil occurrence records and associated (palaeo) environmental data. Our research is the first to collate the fossil and historic distribution records of the wildcat in Britain and to analyse them in combination with contemporary records, in order to establish a modern conservation context. To this end, we hypothesise that the Scottish population of wildcats is environmentally truncated and that reliance on this restricted modern distribution data limits the scope for conserving the species in Britain. We predict that incorporating the wildcat's fossil and historic records into habitat suitability models (HSMs) will increase the amount of environmentally suitable habitat predicted across Britain, predominantly in England and Wales. We further predict that including the wildcat's fossil and historic record will broaden our understanding of the species–environment relationship and enhance the scope of reintroduction projects for wildcats in Britain, particularly outside Scotland.

## 2 Materials and Methods

### 2.1 European Wildcat Occurrence Records

Wildcat occurrence records were collected across Great Britain, including Ireland, for three time periods: modern, historic and fossil (Appendix S1). Modern pseudo‐occurrence records were obtained from the Scottish wildcats IUCN range polygons (available at iucnredlist.org) (Figure 2). These pseudo‐records were generated because modern georeferenced occurrence records of Scottish wildcats are limited, and as the species has undergone recent range contraction, occurrence records pre‐2010 are considered out of date (Gerngross et al. 2023) The IUCN range polygons are constructed by species experts based on the knowledge and available data of species distributions, habitat preferences and ecological limitations and are considered to represent the best probable area of occurrence (IUCN 2018). The Scottish wildcats range polygons were generated in 2022 and represent the most recent estimate of the species probable distribution (Gerngross et al. 2023). Within the range polygons, 300 occurrence records were randomly sampled using the *sf* package in R v.4.2.3, based on the upper limit of the species population estimate of 30–430 mature individuals (Pebesma 2018; Breitenmoser et al. 2019). Historic occurrence records were collected from 1675 to 1970. These records were obtained from published academic research and databases containing historic biodiversity literature for Great Britain, such as wildlife and hunting records. Quaternary fossil records of wildcats were collated through published academic research, museum collections and global databases such as the Palaeobiology Database (PBDB).

Measures were taken to reduce the risk of including erroneous occurrence records into the databases, such as those from feral or domestic cats. Firstly, only historic wildcat occurrences with specific locations and specified identification methods, such as sightings or examinations of the pelage, were included. Wildcats can be distinguished from feral and domestic cats based on specific pelage and fur characteristics (Kitchener et al. 2005). For the fossil records, only wildcat fossils from georeferenced excavation sites in Great Britain, with rigorous stratigraphic and chronological contexts, were included in the database. Species occurrence records, from the past and present, frequently exhibit spatial–temporal biases and uncertainty (Ward 2012; Monsarrat and Kerley 2018; Benson et al. 2021). Modern and historic occurrence records can be spatially biased towards certain regions and environments that are easily accessed by humans as well as proximity to major research institutions (Bean et al. 2011). Fossil records are prone to both spatial and temporal biases, with younger and larger fossils having a higher preservation and detection likelihood (Daru et al. 2017; Crees et al. 2023). To minimise the effects of these biases, all occurrence records were spatially thinned, within their respective climate time periods, by 4 km using the *spThin* package in R, v.4.2.3 (Aiello‐Lammens et al. 2019). This distance was chosen as it represents recent estimates of the wildcat's home range size in Scotland and should therefore reduce the risk of spatial clumping and the effects of oversampled areas (Kilshaw et al. 2015). Additionally, multiple time periods from the early Holocene to the modern period were sampled to maximise the temporal range of the occurrence records. Although older wildcat fossil records were collected for the Middle and Late Pleistocene, only Holocene records were incorporated in the final models. This is because the Holocene records could be precisely temporally matched to high‐resolution palaeoenvironmental data used to construct the HSMs.

### 2.2 Environmental Predictor Variables

A range of bioclimatic, topographic and anthropogenic predictor variables was selected for the HSMs (Table 1). These predictor variables were chosen as they are ecologically meaningful to the wildcat's distribution, representing environmental tolerances or habitat limitations. All the environmental predictor variables were downloaded as raster layers at 5 km grid cells (2.5 arcminute) resolution. Each predictor variable was assessed for collinearity using the *variance inflation factor* (VIF) function via the *R* package *car* v.3.1.2 (Fox and Weisberg 2018). Predictor variables with VIF values > 3 were excluded from the models, as this indicates high multi‐collinearity, which can induce statistical bias and contribute to poor model performance (Craney and Surles 2002).

**TABLE 1:** Environmental predictor variables used in the habitat suitability models.

Type	Time period	Variable	Proxy	Units	Source
Bioclimate	Modern (1970–2000 ad)	BIO17—Precipitation of the driest quarter	Water availability, vegetation type	mm	Worldclim.org
Bioclimate	Modern (1970–2000 ad)	BIO18—Precipitation of the warmest quarter	Water availability, vegetation type	mm	Worldclim.org
Bioclimate	Modern (1970–2000 ad)	BIO19—Precipitation of coldest quarter	Water availability, vegetation type	mm	Worldclim.org
Palaeoclimate	Late‐Holocene (4200–300 years BP)	BIO17—Precipitation of the driest quarter	Water availability, vegetation type	mm	Paleoclim.org
Palaeoclimate	Late‐Holocene (4200–300 years BP)	BIO18—Precipitation of the warmest quarter	Water availability, vegetation type	mm	Paleoclim.org
Palaeoclimate	Late‐Holocene (4200–300 years BP)	BIO19—Precipitation of coldest quarter	Water availability, vegetation type	mm	Paleoclim.org
Palaeoclimate	Mid‐Holocene (8326–4200 years BP)	BIO17—Precipitation of the driest quarter	Water availability, vegetation type	mm	Paleoclim.org
Palaeoclimate	Mid‐Holocene (8326–4200 years BP)	BIO18—Precipitation of the warmest quarter	Water availability, vegetation type	mm	Paleoclim.org
Palaeoclimate	Mid‐Holocene (8326–4200 years BP)	BIO19—Precipitation of coldest quarter	Water availability, vegetation type	mm	Paleoclim.org
Palaeoclimate	Early‐Holocene (11,700–8326 years BP)	BIO17—Precipitation of the driest quarter	Water availability, vegetation type	mm	Paleoclim.org
Palaeoclimate	Early‐Holocene (11,700–8326 years BP)	BIO18—Precipitation of the warmest quarter	Water availability, vegetation type	mm	Paleoclim.org
Palaeoclimate	Early‐Holocene (11,700–8326 years BP)	BIO19—Precipitation of coldest quarter	Water availability, vegetation type	mm	Paleoclim.org
Topographic	All time periods	Elevation	Climate conditions, vegetation type	km	Worldclim.org
Anthropogenic	All time periods	Human Footprint Index	Feral and domestic cat occurrence, human wildcat conflict	Human Impact	Mu et al. (2022)

Three precipitation variables, namely precipitation of the driest quarter (BIO17), precipitation of the warmest quarter (BIO18) and precipitation of the coldest quarter (BIO19), were downloaded from the WorldClim and PaleoClim data sets. These variables were temporally matched to the occurrence records for the following time periods: early‐Holocene (11,700–8326 years before present [BP]), mid‐Holocene (8326–4200 years BP), late‐Holocene (4200–300 years BP) and modern (1970–2000) (Table 1). The modern bioclimatic variables are based on the latest Coupled Model Intercomparison Project Phase 6 (CMIP6) climate projections (Fick and Hijmans 2017) and are used for both the modern and historic occurrence records as no historic climate reconstructions are currently available. The fossil bioclimate variables are from the Community Climate System Model version 3 (CCSM3) (Fordham et al. 2017). These variables represent precipitation levels during the driest, warmest and coldest seasons, which impact water availability and plant productivity, including growth rates. Consistent year‐round precipitation is an important climatic factor for maintaining the mixed temperate forest environment, which is a strong predictor of wildcat occurrence in Scotland (Kilshaw et al. 2016). Elevation is included as a topographic variable that affects local climate conditions and vegetation structure. Elevation is considered a limiting factor for Scottish wildcats, as well as populations in Europe, which are reported to avoid high elevations during winter and prolonged periods of snowfall (Weber 2018; Anile et al. 2019; Cushman et al. 2024). Elevation was downloaded from Worldclim.org and assumed constant for all time periods. Temperature variables were not included in the models as the relatively mild climate in Britain is within the species thermal tolerance in Europe (Gerngross et al. 2023).

Human footprint data were downloaded from Mu et al. (2022) for the modern time period. This raster layer is comprised of six anthropogenic pressure variables: built environments, population density, nighttime lights, crop and pasture lands, roads and railways and navigable waterways. The human footprint values range from true wilderness (< 1), intact areas (1–4) and modified regions (> 4) with 4–20 indicating medium impact and 20–50 high impact. This predictor variable is a proxy for feral and domestic cat occurrence, which is known to impact wildcat distribution negatively (Kilshaw et al. 2015). Feral and domestic cats are most likely to inhabit areas close to human occupation, with feral cats tending to stay within 3 km of farm or rural buildings to scavenge prey (Kilshaw et al. 2016). The human footprint variable is also associated with the risks of human wildcat conflict, such as roads, traffic and built‐up areas, all of which further negatively impact wildcat habitat suitability. The human footprint data were used for both modern and historic time periods, due to the non‐availability of historic reconstructions. For fossil time periods, the raster cell values were converted to 0, reflecting negligible impacts of humans on the environment during these periods.

### 2.3 Habitat Suitability Models

#### 2.3.1 MaxEnt

Predicted habitat suitability for the wildcat in Great Britain and Ireland was modelled using Maximum Entropy (MaxEnt). MaxEnt is a non‐parametric correlative machine learning algorithm, which employs a presence‐background approach to characterise the probable distribution of a species from environmental data (Phillips et al. 2006; Pearson et al. 2007). A commonly used tool within ecology, MaxEnt was chosen due to its superior performance against other presence‐only machine learning methods and execution with small spatially constrained data sets (Elith 2010; Jarvie et al. 2021). The principle of MaxEnt is to estimate probable distribution by contrasting the distribution of presence records and background points along an environmental gradient (Gomes et al. 2018). The algorithm calculates two probability densities against the environmental predictor variables, one for the background points and the other for the presence records. The probability density of the background points identifies the available environment in the study region, while the probability density of the presence records characterises the environment of the species' occurrence (Li et al. 2011).

#### 2.3.2 Model Construction

HSMs were generated through MaxEnt, using the *maxnet* and *glmnet* packages in *R* v.4.4.1 (Phillips 2017), following the methodology proposed by Mills et al. (2024) (Appendix S2). Wildcat occurrence (presence) records and environmental predictor variables were loaded into *R* and 10,000 background points were generated for each time period, as per MaxEnt's default settings (Liu et al. 2005). Background points were generated within a 200 km buffer zone, cropped to the extent of Britain, around the occurrence records for each time period. This buffer zone was selected to include environments that are assumed accessible to the wildcat through dispersal. MaxEnt uses the background records from the buffer zone to compare the environmental conditions of the occurrence records with those of the wider accessible environment and form suitability projections. Background records were generated using the *raster* and *sp* packages (Hijmans et al. 2025; Pebesma et al. 2025).

Three HSMs were constructed for the wildcat in Great Britain and Ireland (Table 2). These models are (1) *modern only*, using modern occurrence records and environmental variables, (2) *modern historic*, using modern and historic occurrence records and environmental variables and (3) *modern fossil*, using modern, historic and fossil occurrence records and associated (palaeo)environmental variables. Each of the three models was then projected onto current climate conditions.

**TABLE 2:** The three habitat suitability models constructed for wildcat in Great Britain and Ireland.

Model	Occurrence records	Predictor variables
Modern only	Modern	Modern climate (BIO17, BIO18, BIO19) Elevation Human Footprint Index
Modern historic	Modern Historic	Modern climate (BIO17, BIO18, BIO19) Elevation Human Footprint Index
Modern fossil	Modern Historic Late‐Holocene Mid‐Holocene Early‐Holocene	Modern climate (BIO17, BIO18, BIO19) Late‐Holocene climate (BIO17, BIO18, BIO19) Mid‐Holocene climate (BIO17, BIO18, BIO19) Early‐Holocene climate (BIO17, BIO18, BIO19) Elevation Human Footprint Index

Habitat suitability maps were generated for each model, displaying MaxEnt's relative index of predicted environmental suitability across geographic space. The index is continuous, with values ranging from 0 to 1, and are interpreted as five categories of environmental suitability: poor suitability (< 0.2), low (0.2–0.4), moderate (0.4–0.6), good (0.6–0.8) and excellent (> 0.8) (Ardestani et al. 2015). The suitability index was not converted into a binary threshold of suitable and unsuitable areas, due to the subjectivity of selecting arbitrary cut‐off values, and the loss of environmental suitability gradients, which is important for conservation applications (Calabrese et al. 2013; Scherrer et al. 2019; Warren et al. 2019). Response curves and a jackknife test, evaluating the importance of each environmental predictor variable on model predictions, were generated using the *cloglog* function from the *glmnet* package and *precrec* package, respectively (Saito and Rehmsmeier 2017; Friedman et al. 2023). Response curves test the influence of each environmental predictor variable on the suitability projections whilst the other variables are held constant. The jackknife test, leave one variable out, evaluates the importance of each variable by comparing the model's performance with each variable in isolation and with each variable excluded in turn.

#### 2.3.3 Model Performance

Spatial blocking was applied to the models to account for spatial autocorrelation and facilitate predictive performance testing (Elith and Leathwick 2009). The occurrence and environmental data for each model was divided into a training data set (75%) to create the model and testing data set (25%) to assess model performance, using the *R* package *blockcv* (Valavi et al. 2018). Four statistical tests were applied to the models: Area Under the Curve (AUC), Continuous Boyce Index (CBI), Minimum Training Presence (ORMTP) and 10% Omission Rate (OR10).

The AUC is a threshold measure of predictive accuracy, indicating how well the model can predict random locations from the dataset (Hoffman et al. 2017). AUC values are categorised as excellent (> 0.9), good (0.8 < AUC ≤ 0.9), acceptable (0.7 < AUC ≤ 0.8), bad (0.6 < AUC ≤ 0.7) and invalid (0.5 < AUC ≤ 0.6) (Ardestani et al. 2015). The CBI also assesses predictive accuracy, with values ranging from −1 for counter predictions, 0 for random predictions and +1 for predictions consistent with testing data (Manzoor et al. 2018). An omission test, Minimum Training Presence (ORMTP) was used to test the models for overfitting. Overfitting occurs when machine learning models are fit too closely to the training dataset and describe random errors rather than environmental relationships (Boria et al. 2014). Omission values close to or equal to zero indicate minimal overfitting and accurate model predictions (Radosavljevic and Anderson 2014).

## 3 Results

**FIGURE 1:**
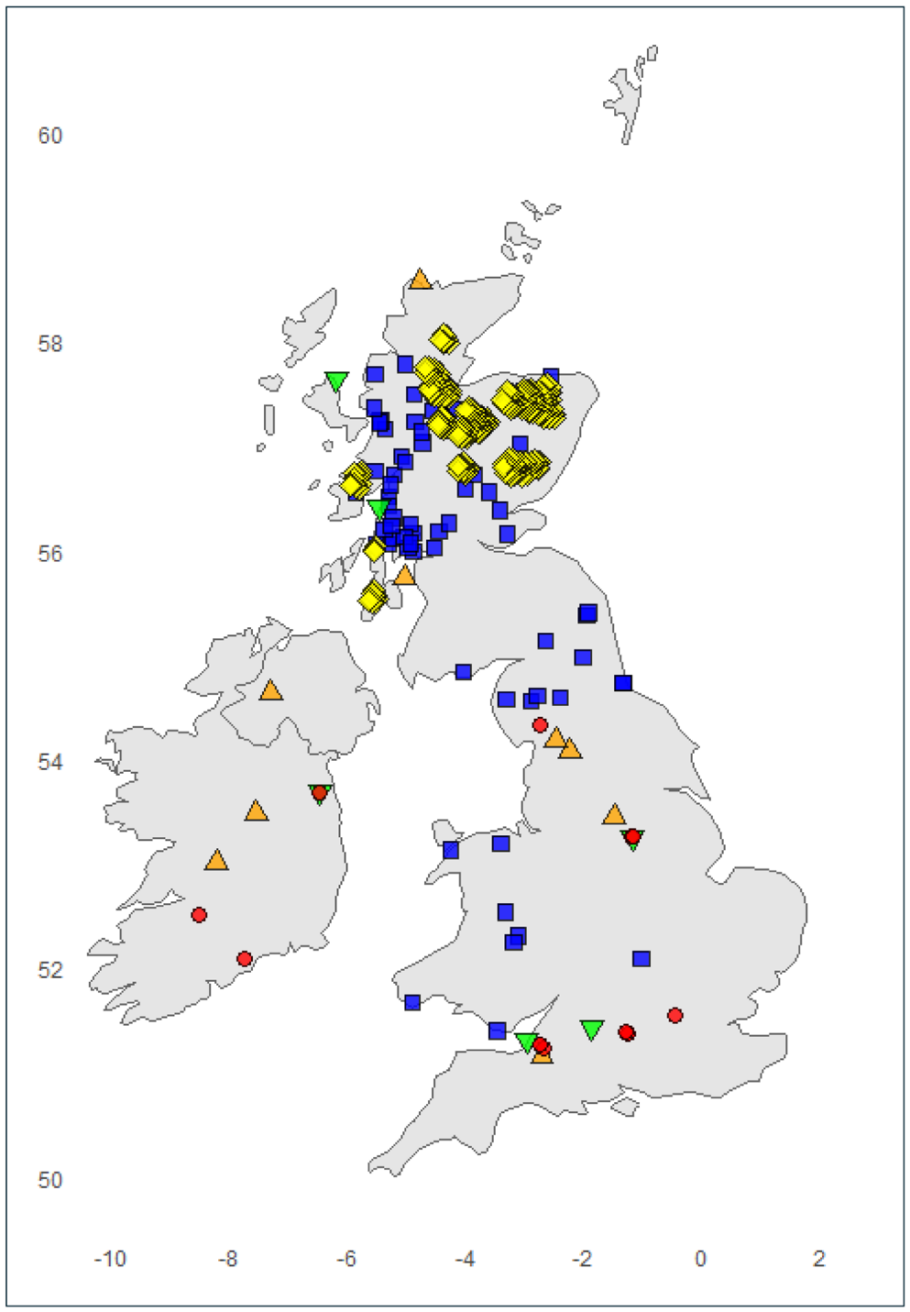
The wildcat occurrence records from Great Britain and Ireland used to construct the habitat suitability models. Occurrence records were obtained for the following time periods: modern (yellow diamonds), historic (blue squares), late‐Holocene (orange triangles), mid‐Holocene (green triangles) and early‐Holocene (red circles).

Wildcat occurrence records were obtained from global databases and published literature for modern, historic and fossil time periods. Overall, 28 fossil and 66 historic records were obtained, and 300 modern records were generated. After spatially thinning the datasets by 4 km, 24 fossil, 62 historic and 163 modern records were used in the models (Figure 1). The fossil records are all Holocene in age and dated to the following time periods: nine from the late‐Holocene (4200–300 years BP), six from the mid‐Holocene (8326–4200 years BP) and nine from the early‐Holocene (11,700–8326 years BP). The modern records are located exclusively in Scotland, whereas the historic records are distributed in Scotland, as well as northern and central England, and Wales. The fossil records are more widely distributed across Scotland, northern, central and southern England, and Ireland.

The predictive accuracy of the HSMs was assessed through AUC and CBI tests. AUC values of 0.87 for the modern only, 0.85 for modern historic and 0.89 for the modern fossil model indicate good predictive accuracy across all three models (Table 3). In addition, CBI values close to 1 for all three models demonstrate model predictions that are consistent with the actual occurrence records. The omission values are also close to 0 for all models, which suggests the models are not overfit to the training data. Overall, the modern fossil model had the highest AUC and CBI values and the lowest ORMPT and standard deviations, indicating better predictive accuracy and model performance compared to the modern only and modern historic models.

**TABLE 3:** Model performance metrics and standard deviations for the three habitat suitability models for wildcat in Great Britain and Ireland.

Model	AUC	SD	CBI	SD	ORMTP	SD
Modern only	0.87	0.039	0.969	0.009	0.112	0.021
Modern historic	0.85	0.079	0.981	0.005	0.101	0.014
Modern fossil	0.89	0.029	0.991	0.004	0.013	0.004

**FIGURE 2:**
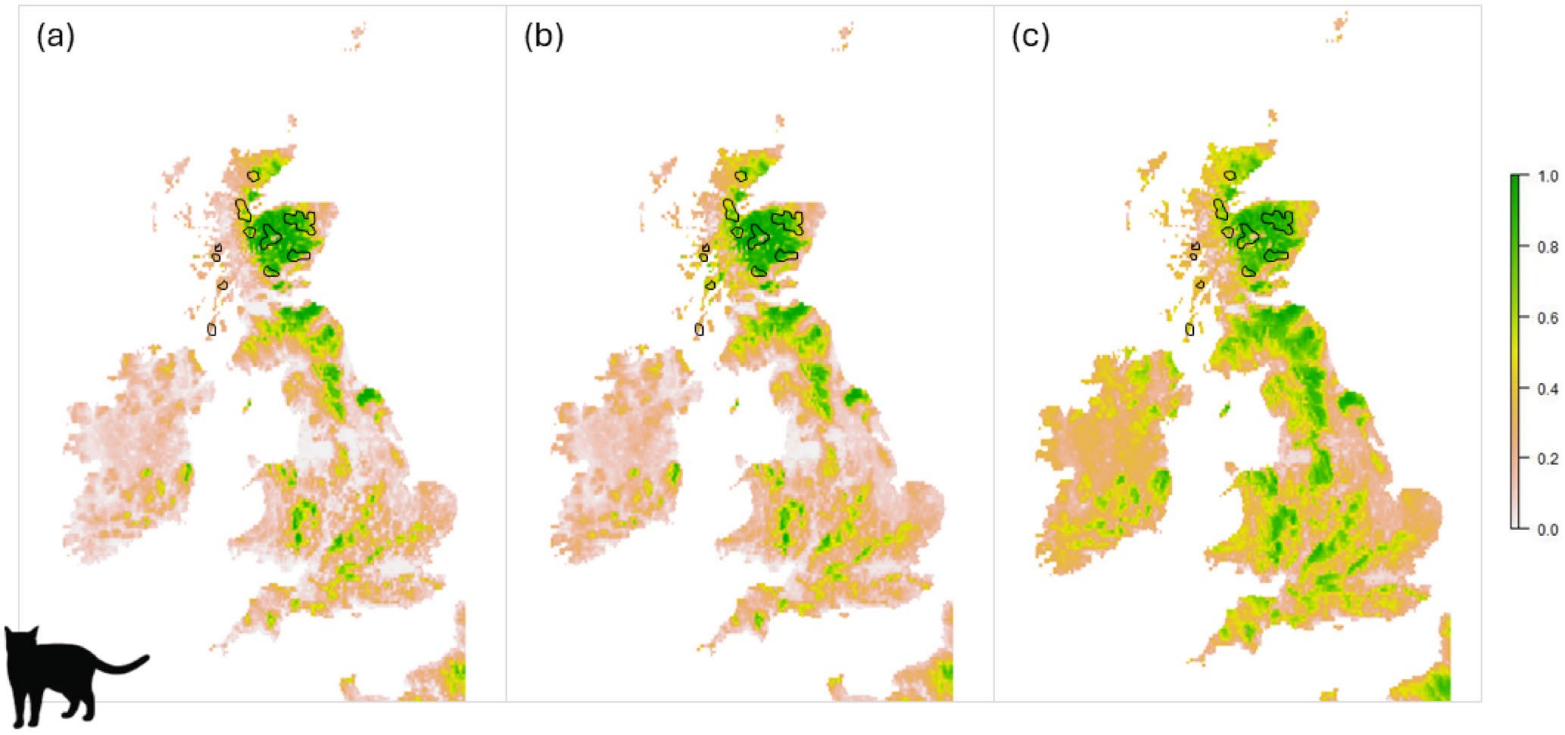
Habitat suitability models of Great Britain and Ireland for the European wildcat under current climate conditions, using the following occurrence records and associated (palaeo) environmental variables: (a) modern only, (b) modern historic, (c) modern fossil. The scale represents predicted environmental suitability, with 1 representing highly suitable environments and 0 representing poor suitability environments. Red polygons represent the wildcat's probable Scottish range from the IUCN range polygons (Gerngross et al. 2023). Black polygons are national park boundaries for England, Wales and Scotland. 1, Dartmoor; 2, Exmoor; 3, New Forest; 4, South Downs; 5, The Broads; 6, Peak District; 7, North York Moors; 8, Yorkshire Dales; 9, Lake District; 10, Northumberland; 11, Loch Lomond and The Trossachs; 12, Cairngorms; 13, Snowdonia; 14, Brecon Beacons (Bannau Brycheiniog).

Under current climate conditions, the modern only model predicted suitable (> 0.6) wildcat habitat areas in Scotland, England, Wales, and very small areas of Ireland (Figure 2a). The largest predicted habitat area is in central Scotland, encompassing the Grampian Mountains and Cairngorms National Park (Figure 2a). A large area of the Scottish Southern Uplands is also predicted suitable, as well as some small areas of the Northern Uplands. In England, suitable predicted habitat is mainly in the north, with smaller, less suitable areas distributed across central and southern England. The largest suitable areas are in Northumberland and Yorkshire, inclusive of the North York Moors and Yorkshire Dales National Parks. In Wales, there are several small suitable areas predicted in the north and central regions, including a small area of the Brecon Beacons (Bannau Brycheiniog) National Park.

The addition of historic records into the modern historic model increased the amount of predicted excellent (> 0.8) and good (> 0.6–0.8) habitat areas by 12.2% and 23.6%, respectively (Table 4). Although the geographical distribution of predicted suitable habitat remains similar to that of the modern only model, the increase includes some very small, fragmented areas of western Scotland, as well as further habitat in the Southern Uplands, Yorkshire Dales and Dartmoor National Park (Figure 2b). There is also an increase of moderate (> 0.4–0.6) and poor (> 0.2–0.4) habitat areas predicted, 38.1% and 34.1%, respectively, and a decrease in poor suitability habitat (< 0.2) by 19% (Table 4).

**TABLE 4:** Amount of suitable habitat area in km^2^ for the three models (modern only, modern historic and modern fossil).

Habitat suitability	Modern only	Modern historic	Modern fossil
Excellent (> 0.8)	12,007.38	13,474.60	16,470.29
Good (> 0.6 to < 0.8)	13,043.06	16,116.40	38,185.77
Moderate (> 0.4 to < 0.6)	25,935.60	35,839.97	80,874.44
Low (> 0.2 to < 0.4)	63,430.96	85,027.73	124,684.5
Poor (< 0.2)	189,507.10	153,464.90	43,709.61

In contrast, the inclusion of wildcat fossil records and associated palaeoenvironmental variables into the modern fossil model has a profound effect on the predicted amount of excellent and good habitat when compared to the modern only model, increasing it by 37.2% and 192.7%, respectively (Table 4). The predicted suitable habitat areas include the extent of the modern only and modern historic models, as well as capturing a significantly larger and continuous area of southern Scotland and northern England, encompassing the North York Moors, Yorkshire Dales, Lake District and Northumberland National Parks (Figure 2c). New suitable areas for wildcat are predicted in central and southern England, including the Peak District and Exmoor National Park. An increase in suitable areas is also observed across Wales, including small areas of Snowdonia and the Brecon Beacons. The predicted amount of moderate and poor habitat increased by 211% and 96.6%, respectively, and poor suitability habitat decreases by 76.9% (Table 4).

**FIGURE 3:**
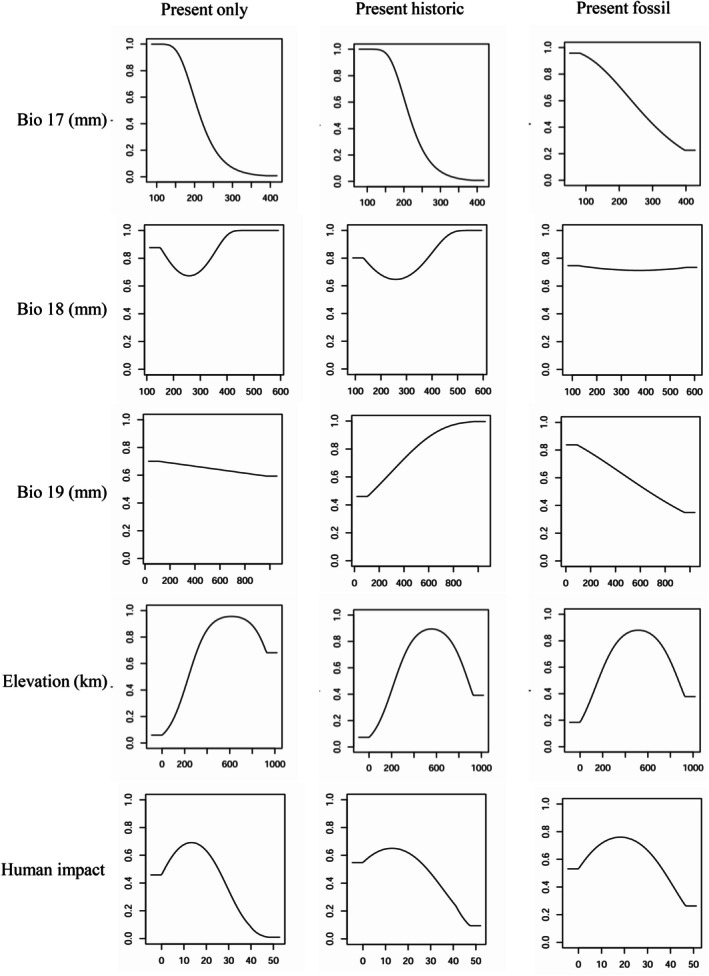
Response curves for the three habitat suitability models.

The response curves show the different thresholds for each environmental predictor variable in predicting suitable (> 0.6) habitat conditions for the modern only, modern historic and modern fossil models (Figure 3). The addition of historic records into the modern historic model altered the threshold for suitable habitat conditions for BIO19 (precipitation of the coldest quarter) from 0–800 mm in the modern only model to 250–1000 mm and elevation from 250–1000 to 250–850m. The suitability thresholds for BIO17 (precipitation of the driest quarter) and human impact do not vary between the modern only and modern historic models. In comparison, the addition of historic and fossil records into the modern fossil model increased the suitable habitat threshold for BIO17 from 100–200 mm in the modern only model to 100–250 mm and human impact from 4–22 to 4–33. BIO19 and elevation are reduced from 0–800 to 0–500 mm and from 250–1000 to 200–820 m, respectively. The suitability threshold for BIO 18 (precipitation of the warmest quarter) remained stable, 100–600 mm, for all three models.

**FIGURE 4:**
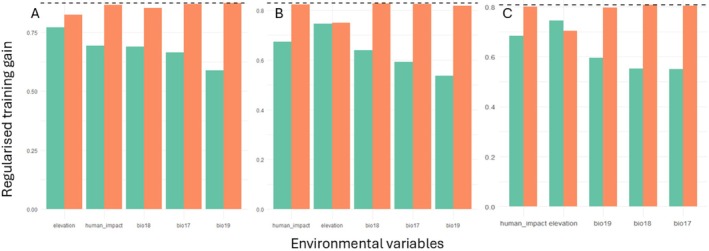
Jackknife test of relative variable importance for the (A) modern only, (B) modern historic and (C) modern fossil models. Green bars are regularised training gain with only the variable, and orange bars are regularised training gain without the variable.

The results of the jackknife test demonstrate that elevation is the most important factor in predicting wildcat environmental suitability, followed by human impact for all three models (Figure 4). For the modern only and modern historic models, BIO18 is the third most important variable for environmental suitability, with BIO17 and BIO19 being the least important. In contrast, BIO19 is the third most important variable for the modern fossil model, with BIO18 and BIO17 being the least important.

## 4 Discussion

On account of persecution and habitat destruction, British wildcat populations are now located exclusively in Scotland, where conservation efforts are accordingly predominantly focused (Breitenmoser et al. 2019). However, the past and present wildcat distribution records presented here demonstrate that historic and Holocene fossil populations of wildcats once had a much broader distribution throughout Britain (Figure 1). Here, we discuss the results of the models in terms of wildcat range and niche dynamics, potential reintroduction opportunities and the wider importance of these models to endangered species conservation.

### 4.1 Range and Niche Dynamics

The inclusion of fossil and historic occurrence records into the models increased the amount and extent of predicted suitable wildcat habitat across Britain (Figure 2; Table 4). This is attributed to the wider distribution of the wildcat's fossil and historic records across Scotland, England, Wales and Ireland compared to the species modern range in Scotland alone, and therefore incorporating a broader range of environmental conditions into the model (Figure 1). This altered certain model parameters determining wildcat environmental suitability, leading to more diverse habitats being predicted suitable (Figure 3). Specifically, good to excellent suitability for BIO17, precipitation of the driest quarter, increased from 200 to 250 mm, and elevation decreased from 250 to 200 m. Therefore, areas with higher rainfall and lower elevation are predicted suitable in the modern fossil model (Figure 2). This is further supported by the results of the jackknife test, which identified elevation as the most important variable for suitability predictions in the models (Figure 4). This is consistent with understandings of contemporary wildcat ecology, with lower elevations, > 650 m, and climate types that promote a mixture of grass and woodland considered favourable habitat conditions for wildcats (Kilshaw et al. 2016; Breitenmoser et al. 2019). In contrast, areas without grassland and high elevations are associated with wildcat absence (Silva, Rosalino, et al. 2013), thereby underlining the forced occupation of higher ground by Scottish wildcats in the face of habitat loss and persecution despite lower environmental suitability. Indeed, human impact was identified as the second most important suitability predictor in the models, with increasing human footprint negatively correlated with environmental suitability (Figures 3 and 4). The broader environmental thresholds from the modern fossil model therefore suggest that Holocene fossil and historic wildcat populations occupied a wider range of environmental conditions and had a greater realised niche breadth than the modern Scottish population.

In contrast, the modern only model, constructed with modern wildcat occurrence records and associated environmental variables from Scotland only, predicted the lowest amount of good to excellent suitability (> 0.6) and the highest amount of poor suitability (< 0.2) habitat across Britain (Figure 2). This is due to the narrower environmental parameters used in model calibration to characterise suitable wildcat habitat, compared to the modern historic and modern fossil models (Figure 3). These parameters are derived from the wildcat's contemporary range, which is restricted to fragmented regions of northern, central eastern and western Scotland (Davis and Gray 2010). Consequently, the main areas predicted as suitable lie within the species' current range estimate, which includes areas of Moray, Inverness, Aberdeenshire and Angus, where there are known populations of genetically pure wildcats (Gerngross et al. 2023). The wildcat's modern range is spatially and ecologically reduced compared to historic and Holocene fossil populations, with the species absent from large portions of its former range. Known as species–environment truncation, the wildcat is occupying an impoverished set of environmental conditions that does not reflect the species' full environmental relationship (Faurby and Araújo 2018). If left undetected, truncation leads to incomplete and biased representations of the species' environmental niche and ecological tolerances within HSMs, as well as underestimating suitable environment areas (Bush et al. 2018; Goicolea et al. 2024; Mills et al. 2024).

Including historic records into HSMs can reduce the effect of species–environment truncation on model predictions by including environmental conditions that the species no longer occupies but which remain environmentally suitable (Chatterjee et al. 2012; Monsarrat et al. 2019). The inclusion of historic wildcat data into the models led to a small increase in good to excellent habitat predicted and a decrease in poor suitability habitat compared to the modern only model (Figure 2b; Table 4). This is attributed to the historic records altering some of the suitability thresholds of the predictor variables including BIO19, precipitation of the coldest quarter, and elevation (Figure 3). Consequently, along with the species' modern range, areas with slightly higher (< 1000 mm) rainfall in the coldest quarter and lower elevations (< 850 m) in northern and southern England are also predicted suitable (Figure 2b). However, the modern historic model also predicted less good to excellent suitability and more poor suitability habitat than the modern fossil model, indicating that historic wildcat populations were also experiencing range contraction and restricted environmental conditions when compared to Holocene fossil populations (Cromsigt et al. 2012; Faurby and Svenning 2015). It is well documented that wildcats have suffered extensive anthropogenic persecution and regional extinctions across Britain for hundreds of years, and therefore, their historic record is unlikely to capture the species' full environmental relationship (Breitenmoser et al. 2019). This further highlights the importance of including deeper‐time fossil records into HSMs to better represent species' environmental tolerances and niche breadth prior to intense anthropogenic pressures (Jarvie et al. 2021; Crees et al. 2023; Mills et al. 2024).

### 4.2 Conservation Opportunities

Our results have implications for the wildcat's refugee status in Scotland. The refugee concept describes species that have become marginalised in suboptimal habitat conditions through human pressures, climate change and habitat loss, and which are then mistakenly conserved in that habitat due to conservationists perceiving it to be optimal (Cromsigt et al. 2012; Kerley et al. 2012). The wildcat's modern range in northern and central eastern Scotland, including areas of the Black Isle, Inverness, Cairngorms and Aberdeenshire, is predicted to be environmentally suitable in all three models (Figure 2; Davis and Gray 2010; Breitenmoser et al. 2019, and references therein). This suggests that wildcats have occupied these conditions from fossil to modern populations and that this habitat therefore aligns with the species‐environment relationship and fundamental niche (Maguire et al. 2015). If central and eastern Scottish wildcat populations were refugees, the addition of historic and fossil environmental data into the models would have lowered the suitability of these areas (Mills et al. 2024). Our results therefore support in situ conservation efforts within wildcat strongholds in northern and central‐eastern Scotland, which focus on preserving genetically pure individuals and strengthening existing wildcat populations through wild releases and threat reduction (Breitenmoser et al. 2019). Here, recent successes include the work of the European partnership project Saving Wildcats, which released 19 wildcats into the Cairngorms National Park in 2023, and in 2024, recorded via camera trap, the first wild‐born kittens in the release site (Saving Wildcats 2024).

However, the wildcat's range in western Scotland, including the conservation priority area of Morvern, is predicted as low to poor habitat in all three models (Figure 2). This suggests that the environmental conditions in western Scotland are suboptimal and lie on the outskirts of the wildcat's suitable environmental range and occupied niches for past and present populations. Current wildcat abundance is low in western Scotland, with populations highly fragmented and declining in size (Davis and Gray 2010). Wildcat density estimates are 1 cat/100 km^2^ in the west compared to 68 cats/100 km^2^ in the north‐east (Breitenmoser et al. 2019). The decline in the west has been attributed to low prey densities, especially of the European rabbit; however, our results suggest it could be an underlying abiotic environmental factor causing suboptimal habitat conditions, such as elevation, which was the most important variable for suitability predictions in all three models (Breitenmoser et al. 2019). This supports existing research by Cushman et al. (2024), a study that identified elevation as an important limiting factor to Scottish wildcat distribution and predicted low suitability in open high elevation areas, as well as those with high human occupation. Wildcat populations were restricted to north‐west Scotland towards the end of the 19th century due to continued persecution and habitat loss (Langley and Yalden 1977). Easterbee et al. (1991) suggested that the wildcat may have survived in this region due to the remoteness and lower human population density and persecution risk, rather than high habitat suitability. Our results support this statement, as the human impact variable is identified as the second most influential for the model's suitability predictions, and the response curves demonstrate that habitat suitability decreases as human footprint increases (Figures 3 and 4). We consequently propose that the wildcat populations in western Scotland are refugees inhabiting suboptimal environmental conditions.

The models presented here also predicted environmentally suitable areas across the wildcat's historic and fossil range, where the species is currently absent due to anthropogenic extirpations. A large continuous area of southern Scotland and northern England is predicted suitable, encompassing the North York Moors, Yorkshire Dales, Lake District and Northumbria National Parks (Figure 2c). Areas of northern, central and eastern Wales are also predicted suitable, as well as regions of central and southern England, including Exmoor and Dartmoor National Parks. Persecution and habitat loss were the main reasons for the wildcat's historic population decline in Scotland and disappearance from England and Wales, with records of the species being hunted for sport during mediaeval times (Langley and Yalden 1977; Easterbee et al. 1991). Feasibility and strategic studies for the potential reintroduction of wildcats into Wales and England are currently being developed by various academic and conservation organisations (Macpherson et al. 2020). For example, the Southwest Wildcat Project is a cross‐sectoral partnership investigating the possible return of wildcats to the Southwest of England (Devon Wildlife Trust 2024).

Facilitating the release and recolonisation of wildcats across their former natural, pre‐anthropogenically impacted range will not only strengthen the stability of existing populations and reduce extinction risk but also help to restore native biodiversity and lost ecological functions across Britain (Brown et al. 2011; Wilson and Campera 2024). As a meso‐predator, wildcats are effective regulators of prey species populations and can provide trophic cascades by influencing species at multiple trophic levels (Tossens et al. 2024). Furthermore, wildcats, like other wild felid species, can serve as indicators of ecosystem health, with the species’ presence associated with abundant prey and low human disturbance (Sosa‐López et al. 2023). A recent report by the IUCN SSC Cat Specialist Group concluded that the Scottish population of wildcats is too small for viable recovery and that population reinforcement via the release of continental wildcats into suitable areas is required to prevent extinction in Britain (Breitenmoser et al. 2019). As mentioned above, this could be achieved through introductions from the west‐central European matrilineage (Mattucci et al. 2015), to which all Holocene wildcats in Britain belong (Marr 2016). The results from this study therefore underline the suitability of England and Wales for wildcat reintroductions and propose areas within the two countries that overlap with the boundaries of national parks and protected areas that would offer an appropriate starting point for conservation projects.

However, it must be acknowledged that species reintroductions are extremely complex and carry many potential risks for the target species, other wildlife, domestic animals and human societies (Lewis et al. 2019). Thorough planning and extensive modelling are therefore required to ensure long‐term population viability and environmental suitability (Wilson 2004). For the wildcat in Britain, more complex HSMs are needed that incorporate additional biotic and anthropogenic variables that are absent from this study. For example, prey availability has been identified as a strong habitat suitability influence for wildcat populations in both Scotland and Europe (Oliveira et al. 2018; Breitenmoser et al. 2019). Population declines of the European rabbit and small rodents have reportedly been linked to local extirpation of wildcats, including in the far north of Scotland (Breitenmoser et al. 2019). Another potentially significant variable is the risk of wildcat persecution, particularly from predator control for game hunting. Historically, wildcats were considered pests to many sport hunting estates, and although the species is now legally protected in Britain, there is a risk that they could be mistaken and accidentally shot by gamekeepers controlling feral cats (Breitenmoser et al. 2019). The large area of southern Scotland predicted suitable in this study overlaps with a major area for red grouse hunting, where predators are purposefully kept to a minimum (Figure 2; Macpherson et al. 2020). Understanding the perception of key stakeholders, including local farmers and estate owners, towards wildcat reintroductions in Britain and mapping the potential risk of wildcat persecution within predicted environmentally suitable regions is therefore recommended before any reinforcement or reintroduction projects are considered (Wilson and Campera 2024; Breitenmoser et al. 2019).

One of the most significant threats to current wildcat populations is the risk of hybridisation with feral and domestic cats (Breitenmoser et al. 2019). Hybridisation is threatening the genetic integrity of wildcats, with Macdonald et al. (2004) estimating fewer than 400 ‘pure’ wildcats remained in Scotland. However, it is unknown whether hybridisation was a historic event that occurred after World War 1, associated with the expansion of wildcat populations due to reduced persecution pressure as game keepers were conscripted, or if it is still continuing at high levels today (Easterbee et al. 1991; Kitchener 1992). Our results demonstrate that human impact is the second most significant influence on the model's environmental suitability projections, even with the addition of fossil records. As the human footprint increases, predicted environmental suitability decreases. This supports existing studies that wildcat occupancy is negatively associated with human density and activities, whereas feral and domestic cats are positively associated with human habitation (Kilshaw et al. 2015). Hybridisation risk is considered highest in areas with high pet cat density, low prey populations, and minimal suitable habitat (Kilshaw et al. 2015). Therefore, sites considered suitable for wildcat recovery and reintroduction should be located a considerable distance away from human occupation in hybridisation‐free zones. This will be challenging in southern England, where feral and domestic cat density is much higher than in remote areas of northern England and Scotland, and is an issue that future models will need to address (Breitenmoser et al. 2019). The habitat suitability projections presented in this study, however, provide a new overview of the maximum amount of potentially suitable wildcat habitat available in Britain and the importance of certain environmental variables to suitability projections. These models represent an important step in assessing the reintroduction potential of the species to its pre‐anthropogenic range.

### 4.3 Wider Importance

Our models demonstrate the importance of incorporating past distribution and environmental tolerances into HSMs for species that have undergone extensive anthropogenic range contraction and are suffering environmental truncation (Monsarrat et al. 2019). HSMs calibrated with truncated modern range data alone may present a restricted view of the species' realised niche, the occupied portion of the fundamental niche and fail to capture the full breadth of the species–environment relationship (Maguire et al. 2015; Waterson et al. 2016). The outputs of such HSMs are likely to underestimate or misrepresent the species' ecological tolerances and projected environmental suitability, thereby misguiding and reducing the effectiveness and scope of conservation strategies (Dawson et al. 2011; Lima‐Ribeiro et al. 2017).

By utilising historic and fossil distribution data, previously occupied niches and associated environmental conditions are included in the models to give a better representation of the species' fundamental niche during different climate episodes (Lima‐Ribeiro et al. 2017; Mills et al. 2024). As a consequence, environmental conditions that are no longer occupied by the species, because of anthropogenic range contraction, but which remain environmentally suitable are incorporated into environmental suitability projections. This multi‐temporal calibration approach can improve the predictive performance of HSMs (see Nogués‐Bravo 2009), particularly for future climate change scenarios (Jones et al. 2019). Under such scenarios, portions of a species' fundamental niche that are currently unoccupied may become available; however, HSMs calibrated with restricted modern environmental conditions may not identify these apparently non‐analogue conditions as suitable (Maguire et al. 2015). In addition, multi‐temporal models can be used to validate existing conservation strategies by contributing to the understanding of a species' refugee status, namely whether the current environmental range is optimal and subsequent conservation strategies effective. This is evidenced in this study, and also by Mills et al. (2024) where both the target species' current ranges are predicted to be environmentally suitable, even with the addition of past occupied environmental conditions before anthropogenic range contraction. Through multi‐temporal HSMs, a better understanding of the species' environmental relationship and presently unoccupied portions of the fundamental niche can be obtained in order to provide more accurate long‐term habitat suitability projections and sustainable conservation opportunities.

## 5 Conclusion

Our study demonstrates the importance of including palaeontological and historic data into multi‐temporal HSMs for species that are absent from large portions of their former range and have suffered anthropogenic extirpations and species–environment truncation. Models calibrated with spatially and environmentally restricted modern range data alone risk underestimating the availability of suitable habitat and misinforming subsequent conservation strategies. By incorporating multi‐temporal environmental distributions and data on past occupied niches, models are able to capture the potential environmental range of the species more accurately and to improve the effectiveness of recovery programs. In Britain, the wildcat has experienced extensive range contraction, with the modern Scottish population representing only a portion of the environmental conditions the species could occupy and the western population occupying sub‐optimal environmental conditions. The inclusion of the wildcat's Holocene fossil and historic distribution into the models broadened the environmental thresholds governing suitable habitat, leading to a substantial increase in predicted suitable areas across Scotland, England and Wales. Environmentally suitable areas have been identified for potential wildcat reintroductions, as well as elevation and human impact as significant environmental suitability variables, which are considered important for the species recovery in Britain. However, more complex models incorporating a greater range of biotic and abiotic factors are needed before translocations are considered. Overall, our results suggest that modern wildcat populations in Scotland are occupying only a portion of their potential environmental range in Britain with a restricted realised niche breadth.

## Author Contributions

**Mollie Mills:** conceptualization (lead), data curation (equal), formal analysis (lead), methodology (lead), writing – original draft (lead), writing – review and editing (equal). **Laura Hemmingham:** conceptualization (supporting), data curation (equal), writing – original draft (supporting), writing – review and editing (equal). **Danielle Schreve:** conceptualization (supporting), data curation (equal), writing – original draft (supporting), writing – review and editing (equal).

## Conflicts of Interest

The authors declare no conflicts of interest.

## Supporting information


**Appendix S1:** ece372126‐sup‐0001‐AppendixS1.xlsx.

**Appendix S2:** ece372126‐sup‐0002‐AppendixS2.docx.

## Data Availability

R scripts of the habitat suitability models used in this research are available online via Figshare (https://doi.org/10.17637/rh.28370174). Our database of European wildcat occurrence records from Britain is also provided on Figshare. Modern wildcat occurrence records were obtained from the IUCN Red List assessment for the European wildcat (https://www.iucnredlist.org/), which can be freely downloaded. Modern bioclimate data were obtained from the Wordlclim database (https://worldclim.org/), which can be freely downloaded. Palaeoclimate data was freely downloaded from Palaeoclim (https://palaeoclim.org/).

## References

[ece372126-bib-0001] Aiello‐Lammens, M. , R. Boria , A. Radosavljevic , et al. 2019. Functions for Spatial Thinning of Species Occurrence Records for Use in Ecological Models. CRAN.

[ece372126-bib-0002] Anile, S. , S. Devillard , B. Ragni , F. Rovero , F. Mattucci , and M. Valvo . 2019. “Habitat Fragmentation and Anthropogenic Factors Affect Wildcat *Felis silvestris silvestris* Occupancy and Detectability on Mt. Etna.” Wildlife Biology 2019: 1–13.

[ece372126-bib-0003] Archer, M. , H. Bates , D. Hand , et al. 2019. “The Burramys Project: A Conservationist's Reach Should Exceed History's Grasp, or What is the Fossil Record for?” Philosophical Transactions of the Royal Society B: Biological Sciences 374: 20190221. 10.1098/rstb.2019.0221 PMC6863488 31679491

[ece372126-bib-0004] Ardestani, E. G. , M. Tarkesh , M. Bassiri , and M. R. Vahabi . 2015. “Potential Habitat Modeling for Reintroduction of Three Native Plant Species in Central Iran.” Journal of Arid Land 7, no. 3: 381–390.

[ece372126-bib-0005] Bean, W. , K. Stafford , and J. Brashares . 2011. “The Effects of Small Sample Size and Sample Bias on Threshold Selection and Accuracy Assessment of Species Distribution Models.” Ecography 35, no. 8: 250–258.

[ece372126-bib-0006] Benson, R. , R. Butler , R. Close , E. Saupe , and D. Rabosky . 2021. “Biodiversity Across Space and Time in the Fossil Record.” Current Biology 31: 1–12. 34637736 10.1016/j.cub.2021.07.071

[ece372126-bib-0007] Boria, R. A. , L. Olson , S. Goodman , and R. Anderson . 2014. “Spatial Filtering to Reduce Sampling Bias Can Improve the Performance of Ecological Niche Models.” Ecological Modelling 275: 73–77.

[ece372126-bib-0008] Breitenmoser, U. , T. Lanz , and C. Breitenmoser‐Würsten . 2019. Conservation of the Wildcat (Felis silvestris) in Scotland: Review of the Conservation Status and Assessment of Conservation Actvities. IUCN SSC Cat Specialist Group.

[ece372126-bib-0009] Brown, C. , R. McMorran , and M. Price . 2011. “Rewilding—A New Paradigm for Nature Conservation in Scotland?” Scottish Geographical Journal 127, no. 4: 288–314.

[ece372126-bib-0010] Bush, A. , R. A. Catullo , K. Mokany , A. Thornhill , J. T. Miller , and S. Ferrier . 2018. “Truncation of Thermal Tolerance Niches Among Australian Plants.” Global Ecology and Biogeography 27: 22–31.

[ece372126-bib-0011] Calabrese, J. , G. Certain , C. Kraan , and C. Dormann . 2013. “Stacking Species Distribution Models and Adjusting Bias by Linking Them to Macroecological Models.” Global Ecology and Biogeography 23, no. 1: 99–112.

[ece372126-bib-0012] Campbell, R. D. , M. J. Gaywood , and A. C. Kitchener . 2023. Scottish Wildcat Action: Final Summary Report. NatureScot.

[ece372126-bib-0013] Chatterjee, H. , J. Tse , and S. Turvey . 2012. “Using Ecological Niche Modelling to Predict Spatial and Temporal Distribution Patterns in Chinese Gibbons: Lessons From the Present and the Past.” Folia Primatologica 83: 85–99. 10.1159/000342696 23038160

[ece372126-bib-0014] Corbett, L. K. 1979. “Feeding Ecology and Social Organization of Wild Cats (*Felis silvestris*) and Domestic Cats (*Felis catus*) in Scotland.” PhD thesis, Aberdeen.

[ece372126-bib-0015] Craney, T. , and J. Surles . 2002. “Model‐Dependent Variance Inflation Factor Cutoff Values.” Quality Engineering 14, no. 3: 391–403.

[ece372126-bib-0016] Crees, J. , V. A. Oxley , D. S. Schreve , and S. T. Turvey . 2023. “Challenges for Incorporating Long‐Term Baselines Into Biodiversity Restoration: A Case Study of the Dalmatian Pelican ( *Pelecanus crispus* ) in Britain.” Ibis 165: 365–387.

[ece372126-bib-0017] Cromsigt, J. , G. Kerley , and R. Kowakzyk . 2012. “The Difficulty of Using Species Distribution Modelling for the Conservation of Refugee Species—The Example of European Bison.” Diversity and Distributions 18: 1253–1257.

[ece372126-bib-0018] Cushman, S. , K. Kilshaw , Z. Kaszta , R. Campbell , M. Gaywood , and D. MacDonald . 2024. “Variable Importance and Scale of Influence Across Individual Scottish Wildcat Hybrid Habitat Models.” Ecological Modelling 491: 1–17.

[ece372126-bib-0019] Daniels, M. , M. A. Beaumont , P. J. Johnson , D. Balharry , D. W. MacDonald , and E. Barratt . 2001. “Ecology and Genetics of Wild‐Living Cats in the North‐East of Scotland and the Implications for the Conservation of the Wildcat.” Journal of Applied Ecology 1: 146–161.

[ece372126-bib-0020] Daru, B. , D. Park , R. Primack , C. Willis , and D. Barrington . 2017. “Widespread Sampling Biases in Herbaria Revealed From Large‐Scale Digitization.” New Phytologist 217, no. 2: 939–955. 29083043 10.1111/nph.14855

[ece372126-bib-0021] Davis, A. , and D. Gray . 2010. “The Distribution of Scottish Wildcats (*Felis silvestris*) in Scotland (2006–2008).” Scottish Natural Heritage Commissioned Report.

[ece372126-bib-0022] Dawson, T. , S. Jackson , J. House , I. Prentice , and G. Mace . 2011. “Beyond Predictions: Biodiversity Conservation in a Changing Climate.” Science 332: 53–58. 21454781 10.1126/science.1200303

[ece372126-bib-0023] Devon Wildlife Trust . 2024. “Bringing Wildcats Back.” Devon Wildlife Trust. https://www.devonwildlifetrust.org/.

[ece372126-bib-0024] Di Marco, M. , M. Pacifici , L. Maiorano , and C. Rondinini . 2021. “Drivers of Change in the Realised Climatic Niche of Terrestrial Mammals.” Ecography 44: 1180–1190.

[ece372126-bib-0025] Di Marco, M. , and L. Santini . 2015. “Human Pressures Predict Species' Geographic Range Size Better Than Biological Traits.” Global Change Biology 21, no. 6: 2169–2178. 25504910 10.1111/gcb.12834

[ece372126-bib-0026] Dietl, G. , and K. Flessa . 2011. “Conservation Paleobiology: Putting the Dead to Work.” Trends in Ecology & Evolution 26, no. 1: 30–37. 21035892 10.1016/j.tree.2010.09.010

[ece372126-bib-0027] Easterbee, N. , L. V. Hepburn , and D. J. Jefferies . 1991. Survey of the Status and Distribution of the Wildcat in Scotland, 1983–1987. Nature Conservacy Council for Scotland.

[ece372126-bib-0028] Elith, J. 2010. “The Art of Modelling Range‐Shifting Species.” Methods in Ecology and Evolution 1: 330–342.

[ece372126-bib-0029] Elith, J. , and J. R. Leathwick . 2009. “Species Distribution Models: Ecological Explanation and Prediction Across Space and Time.” Annual Review of Ecology, Evolution, and Systematics 40: 677–697.

[ece372126-bib-0030] Faurby, S. , and M. Araújo . 2018. “Anthropogenic Range Contractions Bias Species Climate Change Forecasts.” Nature 8: 252–256.

[ece372126-bib-0031] Faurby, S. , and J. C. Svenning . 2015. “Historic and Prehistoric Human‐Driven Extinctions Have Reshaped Global Mammal Diversity Patterns.” Diversity and Distributions 21: 1155–1166.

[ece372126-bib-0032] Fick, S. , and J. Hijmans . 2017. “WorldClim 2: New 1 km Spatial Resolution Climate Surfaces for Global Land Areas.” International Journal of Climatology 37, no. 12: 4302–4315.

[ece372126-bib-0108] Fordham, D. A. , F. Saltré , S. Haythorne , et al. 2017. “PaleoView: A Tool for Generating Continuous Climate Projections Spanning the Last 21 000 Years at Regional and Global Scales.” Ecography 40, no. 11: 1348–1358. 10.1111/ecog.03031.

[ece372126-bib-0033] Fox, J. , and S. Weisberg . 2018. An R Companion to Applied Regression. Sage.

[ece372126-bib-0034] Friedman, J. , T. Hastie , R. Tibshirani , et al. 2023. glmnet: Lasso and Elastic‐Net Regularized Generalized Linear Models. CRAN.

[ece372126-bib-0035] Gerngross, P. , H. Ambarli , F. M. Angelici , et al. 2023. “ *Felis silvestris* (Amended Version of 2022 Assessment).” *The IUCN Red List of Threatened Species*.

[ece372126-bib-0036] Goicolea, T. , A. Adde , O. Broennimann , et al. 2024. “Spatially‐Nested Hierarchical Species Distribution Models to Overcome Niche Truncation in National‐Scale Studies.” Ecography 2025: e07328.

[ece372126-bib-0037] Gomes, V. , S. Ijff , N. Raes , et al. 2018. “Species Distribution Modelling: Contrasting Presence‐Only Models With Plot Abundance Data.” Nature 8: 1003. 10.1038/s41598-017-18927-1 PMC5772443 29343741

[ece372126-bib-0038] Grace, M. , H. R. Akçakaya , E. Bennett , et al. 2019. “Using Historical and Palaeoecologicaldata to Inform Ambitious Species Recovery Targets.” Philosophical Transactions of the Royal Society B: Biological Sciences 374: 20190297. 10.1098/rstb.2019.0297 PMC6863500 31679497

[ece372126-bib-0039] Hijmans, R. , J. van Etten , M. Sumner , et al. 2025. Raster: Geographic Data Analysis and Modelling. CRAN.

[ece372126-bib-0040] Hoffman, J. D. , S. Narumalani , D. Mishra , P. Merani , and R. Wilson . 2017. “Predicting Potential Occurrence and Spread of Invasive Plant Species Along the North Platte River, Nebraska.” Invasive Plant Science and Management 1: 359–367.

[ece372126-bib-0041] Howard‐McCombe, J. , D. Ward , A. C. Kitchener , D. Lawson , H. V. Senn , and M. Beaumont . 2021. “On the Use of Genome‐Wide Data to Model and Date the Time of Anthropogenic Hybridisation: An Example From the Scottish Wildcat.” Molecular Ecology 30: 3688–3702. 34042240 10.1111/mec.16000

[ece372126-bib-0042] IUCN . 2018. Mapping Standards and Data Quality for the IUCN Red List Categories and Criteria. IUCN Red List Technical Working Group.

[ece372126-bib-0043] Jarvie, S. , T. Worthy , F. Saltré , R. P. Scofield , P. J. Seddon , and A. Cree . 2021. “Using Holocene Fossils to Model the Future: Distribution of Climate Suitability for Tuatara, the Last Rhynchocephalian.” Journal of Biogeography 48: 1489–1502.

[ece372126-bib-0044] Jones, L. A. , P. D. Mannion , A. Farnsworth , P. J. Valdes , S. J. Kelland , and P. A. Allison . 2019. “Coupling of Palaeontological and Neontological Reef Coral Data Improves Forecasts of Biodiversity Responses Under Global Climate Change.” Royal Society Open Science 6: 182111. 31183138 10.1098/rsos.182111 PMC6502368

[ece372126-bib-0045] Kerley, G. , M. Beest , J. Cromsigt , D. Pauly , and S. Shultz . 2020. “The Protected Area Paradox and Refugee Species: The Giant Panda and Baselines Shifted Towards Conserving Species in Marginal Habitats.” Conservation Science and Practice 2: 1–6.

[ece372126-bib-0046] Kerley, G. , R. Kowalczyk , and J. Cromsigt . 2012. “Conservation Implications of the Refugee Species Concept and the European Bison: King of the Forest or Refugee in a Marginal Habitat?” Ecography 35: 519–529.

[ece372126-bib-0047] Kiessling, W. , W. Raja , V. Roden , S. Turvey , and E. Saupe . 2019. “Addressing Priority Questions of Conservation Science With Palaeontological Data.” Philosophical Transactions of the Royal Society B: Biological Sciences 374: 20190222. 10.1098/rstb.2019.0222 PMC6863486 31679490

[ece372126-bib-0048] Kilshaw, K. 2015. “Introgression and the Current Status of the Scottish Wildcat ( *Felis silvestris silvestris* ).” Unpublished PhD thesis, University of Oxford.

[ece372126-bib-0049] Kilshaw, K. , P. J. Johnson , A. Kitchener , and D. MacDonald . 2015. “Detecting the Elusive Scottish Wildcat *Felis silvestris* Using Camera Trapping.” Oryx 49, no. 2: 207–215.

[ece372126-bib-0050] Kilshaw, K. , R. Montgomery , R. Campbell , et al. 2016. “Mapping the Spatial Configuration of Hybridization Risk for an Endangered Population of the European Wildcat ( *Felis silvestris silvestris* ) in Scotland.” Mammal Research 61: 1–10.

[ece372126-bib-0110] Kitchener, A. 1992. “The Scottish Wildcat (*Felis silvestris*): Decline and Recovery.” In Cats, edited by P. Mansard , 21–41. Ridgeway Trust for Endangered Cats.

[ece372126-bib-0051] Kitchener, A. C. , and T. P. O'Connor . 2010. “Wildcats, Domestic and Feral Cats.” In Extinctions and Invasions: A Social History of British Fauna, edited by T. O'Connor and N. J. Sykes , 83–94. Windgather Press.

[ece372126-bib-0052] Kitchener, A. C. , N. Yamaguchi , J. M. Ward , and D. W. MacDonald . 2005. “A Diagnosis for the Scottish Wildcat ( *Felis silvestris* ): A Tool for Conservation Action for a Critically‐Endangered Felid.” Animal Conservation 8: 223–237.

[ece372126-bib-0053] Klar, N. , N. Fernandez , S. Kramer‐Schadt , et al. 2008. “Habitat Selection Models for European Wildcat Conservation.” Biological Conservation 141: 308–319.

[ece372126-bib-0054] Langley, P. J. , and D. W. Yalden . 1977. “The Decline of the Rarer Carnivores in Great Britain During the Nineteenth Century.” Mammal Review 7, no. 3–4: 95–116.

[ece372126-bib-0055] Lentini, P. , I. Stiremann , D. Stojanovic , T. Worthy , and A. Stein . 2018. “Using Fossil Records to Inform Reintroduction of the Kakapo as a Refugee Species.” Biological Conservation 217: 157–165.

[ece372126-bib-0056] Lewis, J. , A. Tomlinson , M. Gilbert , et al. 2019. “Assessing the Health Risks of Reintroduction: The Example of the Amur Leopard, *Panthera pardus orientalis* .” Transboundary and Emerging Diseases 67: 1177–1188. 31833654 10.1111/tbed.13449

[ece372126-bib-0057] Li, W. , Q. Guo , and C. Elkan . 2011. “Can We Model the Probability of Presence of Species Without Absence Data?” Ecography 34: 1096–1105.

[ece372126-bib-0058] Lima‐Ribeiro, M. , A. Moreno , L. Terribile , and C. Caten . 2017. “Fossil Record Improves Biodiversity Risk Assessment Under Future Climate Change Scenarios.” Diversity and Distributions 23, no. 8: 22–933.

[ece372126-bib-0059] Littlewood, N. A. , R. D. Campbell , L. Dinnie , et al. 2014. “Survey and Scoping of Wildcat Priority Areas.” Scottish Natural Heritage Commissioned Report No. 768.

[ece372126-bib-0060] Liu, C. , P. Berry , T. Dawson , and R. Pearson . 2005. “Selecting Thresholds of Occurrence in the Prediction of Species Distributions.” Ecography 28: 385–393.

[ece372126-bib-0109] Macdonald, D. W. , M. J. Daniels , C. Driscoll , A. C. Kitchener , and N. Yamaguchi . 2004. The Scottish Wildcat: Analyses for Conservation and an Action Plan. Wildlife Conservation Research Unit.

[ece372126-bib-0061] MacDonald, D. W. , N. Yamaguchi , A. C. Kitchener , M. Daniels , K. Kilshaw , and C. Driscoll . 2010. “Reversing Cryptic Extinction: The History, Present, and Future of the Scottish Wildcat.” In The Biology and Conservation of Wild Felids, edited by D. W. MacDonald and A. J. Loveridge , 471–491. Oxford University Press.

[ece372126-bib-0062] Macpherson, J. , S. Carter , S. Devillard , R. Kennerley , S. Ruette , and M. Hudson . 2020. A Preliminary Feasibility Assessment for the Reintroduction of the European Wildcat to England and Wales. Vincent Wildlife Trust.

[ece372126-bib-0063] Maguire, C. , D. Nieto‐Lugilde , C. Fitzpatrick , J. Williams , and J. Blois . 2015. “Modeling Species and Community Responses to Past, Present, and Future Episodes of Climatic and Ecological Change.” Annual Review of Ecology, Evolution, and Systematics 46: 343–368.

[ece372126-bib-0064] Manzoor, S. A. , G. Griffiths , and M. Lukac . 2018. “Species Distribution Model Transferability and Model Grain Size—Finer May Not Always Be Better.” Nature 8: 1–10. 10.1038/s41598-018-25437-1 PMC5940916 29740002

[ece372126-bib-0065] Marr, M. 2016. “Faunal Response to Abrupt Climate Change: The History of the British Mammal Fauna From the Lateglacial to the Holocene.” Unpublished PhD thesis, Royal Holloway University of London.

[ece372126-bib-0066] Martín‐Díaz, P. , J. M. Gil‐Sánchez , E. Ballesteros‐Duperón , et al. 2018. “Integrating Space and Time in Predator–Prey Studies: The Case of Wildcats and Rabbits in SE Spain.” Mammalian Biology 88: 114–122.

[ece372126-bib-0067] Mathews, F. , L. M. Kubasiewicz , J. Gurnell , C. A. Harrower , R. A. McDonald , and R. F. Shore . 2018. “A Review of the Population and Conservation Status of British Mammals: Technical Summary.” A Report by the Mammal Society Under Contract to Natural England, Natural Resources Wales and Scottish Natural Heritage. Natural England.

[ece372126-bib-0068] Mattucci, F. , R. Oliveira , L. Lyons , P. Alves , and E. Randi . 2015. “European Wildcat Populations Are Subdivided Into Five Main Biogeographic Groups: Consequences of Pleistocene Climate Changes or Recent Anthropogenic Fragmentation?” Ecology and Evolution 6, no. 1: 3–22. 26811770 10.1002/ece3.1815 PMC4716505

[ece372126-bib-0069] Mills, M. , D. Schreve , O. Middleton , and C. J. Sandom . 2024. “Going Back for the Future: Incorporating Pleistocene Fossil Records of Saiga Antelope ( *Saiga tatarica* ) Into Global Habitat Suitability Models.” Journal of Biogeography 51, no. 8: 1351–1364.

[ece372126-bib-0070] Monsarrat, S. , and G. Kerley . 2018. “Charismatic Species of the Past: Biases in Reporting of Large Mammals in Historical Written Sources.” Biological Conservation 223: 68–75.

[ece372126-bib-0071] Monsarrat, S. , P. Novellie , I. Rushworth , and G. Kerley . 2019. “Shifted Distribution Baselines: Neglecting Long‐Term Biodiversity Records Risks Overlooking Potentially Suitable Habitat for Conservation Management.” Philosophical Transactions of the Royal Society B: Biological Sciences 374: 1–11. 10.1098/rstb.2019.0215 PMC6863494 31679487

[ece372126-bib-0072] Mu, H. , X. Li , Y. Wen , et al. 2022. “A Global Record of Annual Terrestrial Human Footprint Dataset From 2000 to 2018.” Nature 9, no. 176: 1–9. 10.1038/s41597-022-01284-8 PMC9018937 35440581

[ece372126-bib-0073] Nogués‐Bravo, D. 2009. “Predicting the Past Distribution of Species Climatic Niches.” Global Ecology and Biogeography 18: 521–531.

[ece372126-bib-0074] Oliveira, T. , F. Urra , J. López‐Martín , et al. 2018. “Females Know Better: Sex‐Biased Habitat Selection by the European Wildcat.” Ecology and Evolution 8, no. 18: 9464–9477. 30377515 10.1002/ece3.4442 PMC6194279

[ece372126-bib-0075] Pang, S. , Y. Zeng , J. De Alban , and E. L. Webb . 2022. “Occurrence–Habitat Mismatching and Niche Truncation When Modelling Distributions Affected by Anthropogenic Range Contractions.” Diversity and Distributions 28: 1327–1343.

[ece372126-bib-0076] Pearson, R. G. , C. J. Raxworthy , M. Nakamura , and A. T. Peterson . 2007. “Predicting Species Distributions From Small Numbers of Occurrence Records: A Test Case Using Cryptic Geckos in Madagascar.” Journal of Biogeography 34: 102–117.

[ece372126-bib-0077] Pebesma, E. 2018. “Simple Features for R: Standardized Support for Spatial Vector Data.” R Journal 10, no. 1: 439–446.

[ece372126-bib-0078] Pebesma, E. , R. Bivand , B. Rowlingson , et al. 2025. sp: Classes and Methods for Spatial Data. CRAN.

[ece372126-bib-0079] Phillips, S. 2017. Fitting ‘Maxent’ Species Distribution Models With ‘glmnet’. CRAN.

[ece372126-bib-0080] Phillips, S. J. , R. P. Anderson , and R. E. Schapire . 2006. “Maximum Entropy Modeling of Species Geographic Distributions.” Ecological Modelling 190: 231–259.

[ece372126-bib-0081] Pierpaoli, M. , Z. Birò , M. Hermann , et al. 2003. “Genetic Distinction of Wildcat ( *Felis silvestris* ) Populations in Europe, and Hybridization With Domestic Cats in Hungary.” Molecular Ecology 12, no. 10: 2585–2598. 12969463 10.1046/j.1365-294x.2003.01939.x

[ece372126-bib-0082] Radosavljevic, A. , and R. Anderson . 2014. “Making Better MAXENT Models of Species Distributions: Complexity, Overfitting and Evaluation.” Journal of Biogeography 41: 629–643.

[ece372126-bib-0083] Rodrigues, A. S. , S. Monsarrat , A. Charpentier , et al. 2019. “Unshifting the Baseline: A Framework for Documenting Historical Population Changes and Assessing Long‐Term Anthropogenic Impacts.” Philosophical Transactions of the Royal Society B: Biological Sciences 374: 20190220. 10.1098/rstb.2019.0220 PMC6863499 31679498

[ece372126-bib-0084] Saito, T. , and M. Rehmsmeier . 2017. “Precrec: Fast and Accurate Precision‐Recall and ROC Curve Calculations in R.” Bioinformatics 33, no. 1: 145–147. 27591081 10.1093/bioinformatics/btw570 PMC5408773

[ece372126-bib-0085] Sales, L. P. , M. Galetti , A. Carnaval , S. Monsarrat , J. Svenning , and M. M. Pires . 2022. “The Effect of Past Defaunation on Ranges, Niches, and Future Biodiversity Forecasts.” Global Change Biology 28: 3683–3693. 35246902 10.1111/gcb.16145

[ece372126-bib-0086] Saving Wildcats . 2024. “Wildcats in Britain.” Saving Wildcats. https://www.savingwildcats.org.uk/.

[ece372126-bib-0087] Scheele, B. , C. Foster , S. Banks , and B. Lindenmayer . 2017. “Niche Contractions in Declining Species: Mechanisms and Consequences.” Trends in Ecology & Evolution 32, no. 5: 346–355. 28284374 10.1016/j.tree.2017.02.013

[ece372126-bib-0088] Scherrer, D. , H. Mod , and A. Guisan . 2019. “How to Evaluate Community Predictions Without Thresholding?” Methods in Ecology and Evolution 11, no. 1: 51–63.

[ece372126-bib-0089] Senn, H. V. , M. Ghazali , J. Kaden , et al. 2018. “Distinguishing the Victim From the Threat: SNP‐Based Methods Reveal the Extent of Introgressive Hybridization Between Wildcats and Domestic Cats in Scotland and Inform Future In Situ and Ex Situ Management Options for Species Restoration.” Evolutionary Applications 12: 399–414. 30828363 10.1111/eva.12720 PMC6383845

[ece372126-bib-0090] Silva, A. P. , K. Kilshaw , P. J. Johnson , D. W. MacDonald , and L. M. Rosalino . 2013. “Wildcat Occurrence in Scotland: Food Really Matters.” Diversity and Distributions 19: 232–243.

[ece372126-bib-0091] Silva, A. P. , L. M. Rosalino , P. J. Johnson , D. W. MacDonald , N. Anderson , and K. Kilshaw . 2013. “Local‐Level Determinants of Wildcat Occupancy in Northeast Scotland.” European Journal of Wildlife Research 59: 449–453.

[ece372126-bib-0092] Sommer, R. S. , and N. Benecke . 2006. “Late Pleistocene and Holocene Development of the Felid Fauna (Felidae) of Europe: A Review.” Journal of Zoology 269, no. 1: 7–19.

[ece372126-bib-0093] Sosa‐López, J. , N. Bernal , E. Padilla , and M. Briones‐Salas . 2023. “Analysis of the Effects of Habitat Characteristics, Human Disturbance and Prey on Felids Presence Using Long‐Term Community Monitoring Information.” Nature Conservation 53: 279–295.

[ece372126-bib-0094] Tossens, S. , M. Drouilly , S. Lhoest , C. Vermeulen , and J. Doucet . 2024. “Wild Felids in Trophic Cascades: A Global Review.” Mammal Review 55, no. 1: e12358.

[ece372126-bib-0096] Turvey, S. T. , J. J. Crees , and M. M. Di Fonzo . 2015. “Historical Data as a Baseline for Conservation: Reconstructing Long‐Term Faunal Extinction Dynamics in Late Imperial–Modern China.” Proceedings of the Royal Society B: Biological Sciences 282: 20151299. 10.1098/rspb.2015.1299 PMC4632630 26246553

[ece372126-bib-0095] Turvey, S. , and E. Saupe . 2019. “Insights From the Past: Unique Opportunity or Foreign Country?” Philosophical Transactions of the Royal Society B: Biological Sciences 374: 1–5. 10.1098/rstb.2019.0208 PMC6863498 31679483

[ece372126-bib-0097] Valavi, R. , J. Elith , J. J. Lahoz‐Monfort , and G. Guillea‐Arroita . 2018. “blockCV: An R Package for Generating Spatially or Environmentally Separated Folds for k‐Fold Cross‐Validation of Species Distribution Models.” Methods in Ecology and Evolution 10: 225–232.

[ece372126-bib-0098] van de Velde, S. , E. Jorissen , T. Neubauer , and S. Radan . 2019. “A Conservation Palaeobiological Approach to Assess Faunal Response of Threatened Biota Under Natural and Anthropogenic Environmental Change.” Biogeosciences 16: 2423–2442.

[ece372126-bib-0099] Ward, D. 2012. “More Than Just Records: Analysing Natural History Collections for Biodiversity Planning.” PLoS One 7, no. 11: 1–8. 10.1371/journal.pone.0050346 PMC3504054 23185605

[ece372126-bib-0100] Warren, D. , N. Matzke , and T. Iglesias . 2019. “Evaluating Presence‐Only Species Distribution Models With Discrimination Accuracy is Uniformative for Many Applications.” Journal of Biogeography 47, no. 1: 167–180.

[ece372126-bib-0101] Waterson, A. M. , D. N. Schmidt , P. J. Valdes , et al. 2016. “Modelling the Climatic Niche of Turtles: A Deep‐Time Perspective.” Proceedings of the Royal Society B: Biological Sciences 283: 20161408. 10.1098/rspb.2016.1408 PMC5046900 27655766

[ece372126-bib-0102] Weber, S. 2018. Habitat Model of the European Wildcat ( *Felis silvestris silvestris* ) for the Swiss Jura Mountains, Plateau and Near Pre‐Alpine Regions. Swiss Federal Institute of Technology.

[ece372126-bib-0103] Wilson, C. 2004. “Could We Live With Reintroduced Large Carnivores in the UK?” Mammal Review 34, no. 3: 211–232.

[ece372126-bib-0104] Wilson, S. , and M. Campera . 2024. “The Perspectives of Key Stakeholders on the Reintroduction of Apex Predators to the United Kingdom.” Ecologies 5, no. 1: 52–67.

[ece372126-bib-0105] Yalden, D. 1999. The History of British Mammals. Poyser.

[ece372126-bib-0106] Yamaguchi, N. , A. C. Kitchener , C. Driscoll , and B. Nussberger . 2015. “ *Felis silvestris* . The IUCN Red List of Threatened Species.”

[ece372126-bib-0107] Yang, L. , M. Chen , D. W. Challender , et al. 2018. “Historical Data for Conservation: Reconstructing Range Changes of Chinese Pangolin ( *Manis pentadactyla* ) in Eastern China (1970–2016).” Proceedings of the Royal Society B: Biological Sciences 285: 20181084. 10.1098/rspb.2018.1084 PMC6125891 30135158

