# Supplementary material for: New Perspectives on European Wildcat ( Felis silvestris , Schreber 1777) Habitat Suitability in Britain: Integrating Fossil Records to Improve Baselines for Reintroduction
Source: Ecol Evol. 2025 Sep 25;15(10):e72126. doi: 10.1002/ece3.72126 (PMC12461118; doi:10.1002/ece3.72126)
Supplement: Supplementary file 2 — Appendix S2: ece372126‐sup‐0002‐AppendixS2.docx. [file ECE3-15-e72126-s002.docx]

**Appendix S2** *R* codes for the modern only, modern historic and modern fossil habitat suitability models

**Introduction: Habitat suitability models**

We modelled environmental suitability (climate, topography and human impact) for the European wildcat (*Felis silvestris*) under current climate conditions in Britain, using the maximum entropy algorithm (Maxent) implemented through the *R* package *maxnet.* Maxent is a statistical method for estimating a species probable distribution from geographic occurrence records and associated environmental data. We chose Maxent due to its presence-background approach and superior performance to other modelling techniques, especially with presence-only data. Below, we show the steps we used to run the habitat suitability models (HSMs) with modern only, and modern, historic and fossil occurrence records and associated environmental variables. The models can be projected onto current or future climate change scenarios at the user’s discretion.

1. **Install and load required packages.**

Firstly, we install and load the require packages in *R.* The *pacman* package is a package management tool that combines the functionality of base library functions into intuitively named functions. The packages required are the same for all three model types.

1. **Load environment (predictor) variables**

We used three bioclimate variables, one topographic (elevation) and one human impact variable to characterise suitable environmental conditions for past and present wildcat populations and construct the HSMs. The bioclimatic variables were BIO17, precipitation of the driest quarter, BIO18, precipitation of the wettest quarter, and BIO19, precipitation of the coldest quarter. These environmental variables (listed in Table 1) were chosen as they are ecologically meaningful to the distribution of wildcats and represent some important environmental requirements or limiting factors.

The three bioclimatic variables were downloaded under past and present climate conditions (Table 1). Current climate conditions were downloaded from the WorldClim database (Worldclim.org). Current climate conditions are based on average conditions from 1970 – 2000. We used three different temporal periods of reconstructed past climate conditions from the Holocene, matched to the approximate age of our fossil occurrence records. These time periods were Late-Holocene (4,200 – 300 years BP), Mid-Holocene (8,326 – 4,200 years BP) and Early-Holocene (11,700 – 8,326 years BP) and were downloaded from Paleoclim (Paleoclim.org). Elevation, representing slope and terrain roughness, was downloaded from Worldclim.org. Finally, Human footprint data was downloaded from Mu et al. (2022). This raster layer is comprised of six anthropogenic pressure variables: built environments, population density, nighttime lights, crop and pasture lands, roads and railways, and navigable waterways. The human footprint values range from true wilderness (<1), intact areas (1-4), and modified regions (>4) with 4 – 20 indicating medium impact and 20 – 50 high impact. This predictor variable is a proxy for feral and domestic cat occurrence, which is known to negatively impact wildcat distribution (Kilshaw *et al.,* 2015). Note, in the codes below the environmental variables are listed separately, and it is up to the users discretion to download and use the ones most suitable for their study.

1. **Load and spatially thin species occurrence records**

Modern Scottish wildcat occurrence records (post 1950) were obtained from the International Union for Conservation of Nature (IUCN) red list assessment for the European wildcat In total, 300 presence points were randomly sampled from within the IUCN range polygons for the Scottish wildcat population. Historic and fossil presence records were obtained from the published literature, global databases and museum catalogues (see Appendix S1). Only verified wildcat fossil records, georeferenced to a specific excavation site, and dated either directly or by associated were included in our database.

The presence records were temporally separated and spatially thinned to removed points within 4km of each other. Spatial thinning of presence records is required to reduce the problems associated with spatial sampling bias. Spatial bias may exist where records were obtained, for instance areas with a high number of presence records might not necessarily be the best place for the species in terms of environment. Therefore, we thin presence records to maintain a suitable amount of spatial representation without clumping.

1. **Generate background records and extract the environmental values**

Maxent employs a presence-background approach, where background records are generated to characterize the environment of the study species, whilst the environmental characteristics of the presence points are used to predict the probability of the species presence. To generate the background records, we created and sampled within a 200km buffer zone cropped to the extent of Britain for each temporal period. The buffer zone was selected to include environments that are assumed accessible to wildcat due to their large dispersal capability. We generated 10,000 background records for each time period, as per MaxEnt’s recommended default settings, using the *raster* and *sp* packages.

1. **Spatial blocking for cross block validation**

Spatial blocking was applied to each model to calculate the evaluation statistics needed to assess the model’s predictive performance. Spatial blocking partitions the model into training data, which is used to calibrate the model, and testing data, which is held back to independently test the model performance. Our data was partitioned randomly, with 70% used for calibration and 30% for model testing. We implemented a 5-fold systematic block technique through the *R* package *blockCV* that is recommended for multi-temporal model transfer (Jarvie *et al.,* 2021). Through the 5-fold approach we assessed the model’s predictive performance with two threshold measurements, the area under the curve (AUC) of the receiver operating curve (ROC) plot and Continuous Boyce Index (CBI). Models were assessed for overfitting through two omission rates, minimum training presence (ORMTP) and the 10% omission rate (OR10). Model sensitivity was assessed using the Maximum Sensitivity and Specificity (MSS) test. MSS calculates the threshold at which the sum of the sensitivity (true positive rate) and specificity (true negative rate) is highest.

1. **Create the habitat suitability model**

We now create the habitat suitability model by extracting the folds, with the training and test data, in the buffering object. We employed maxnets default settings, and used the feature class combinations linear, linear quadratic and linear, quadratic and product to avoid overfitting. We used cloglog format to fit the model and predict the distribution of the species. Continuous range maps were produced with the raw habitat suitability values ranging from 0 to 1. Response curves were used to determine the influence of each environmental predictor variable on the model predictions. Response curves were generated by changing one predictor variable whilst the others are held constant. A jackknife test, leave one variable out, was used assess the importance of each variable to the environmental suitability predictions. The jackknife test compares model performance with each variable only, followed by all variables except one.

**Modern only code**

# 1. install and load required packages

install.packages("pacman")

library(pacman)
pacman::p_load(maxnet, rlang, blockCV, dismo, ecospat, precrec, raster,
 sf,tidyverse,rgbif,sp,spThin,maptools,rgeos, rgdal,usdm)

# maxnet is an R package that creates habitat suitability models using Maxent
# rlang is an R package that provides tools for working with core R features and the tidyverse
# blockcv is an R package that creates separate folds for training and testing data for cross-validation
# dismo is an R package that supports habitat suitability models
# ecospat is an R package that supports spatial ecology analysis
# precrec is an R package to calculate ROC curves
# raster is an R package for using rasters
# sf is an R package to implement simple features and encode spatial vector data
# tidyverse is a collection of R packages for data manipulation, exploration and visualization
# rgbif is an R package that allows searching and retrieving data from GBIF
# sp is an R package that provides tools for handling spatial data
# spthin is an R package for spatial thinning of species occurrence records for use in ecological niche models
# maptools is an R package for manipulating spatial data
# rgeos is an R package that performs advanced spatial analysis and manipulation
# rgdal is an R package that allows bindings for spatial analysis, projection and transformation operations
# usdm is an R package that provides functions for exploring the impact of uncertainties on the performance of habitat suitability models

# 2. load environmental (predictor) variables

# current climate data
# bioclimate variables downloaded from worldclim at 2.5 arc second (5km) resolution and crs WGS84
bio17 <- raster("your file directory")# you will need to add your own file directory here where you saved the downloaded climate variables for your species
# name the raster layers after the name of the bioclimatic variable used
# for the wildcat in Britain we used bio 17, 18 and 19
bio18 <- raster("your file directory")
bio19 <- raster("your file directory")


climate <- stack(bio17,bio18,bio19) #stack the current climate variables into a raster stack

slope <- raster ("your file directory") #load your elevation/ topographic variable here
# you will need to add your own file directory here
current <- resample(climate, slope) # use resample to match the geographic extents of the climate stack and slope variable
current <- stack(current, slope) #stack the climate and slope variables into one raster stack
vif(current) # test the variables for collinearity using Variance Inflation Factor (VIF)
# any variable with values <3, indicating high multicollinearity and potential bias, should be excluded from the model


# load HFI data (EPSG: 9001), resample to 2.5 arc minutes, and change crs to WGS 84
hfi <- raster("your file directory")

# stack HFI with climate variables
current <- resample(current, hfi)
climate <- stack(current, hfi)
plot(climate)


# change the names of the raster layers
current_names <- names(climate)
current_names[1] <- "bio17"
current_names[2] <- "bio18"
current_names[3] <- "bio19"
current_names[4] <- "elevation"
current_names[5] <- "human_impact"


# Set the new names to the raster stack
names(climate) <- current_names
plot(climate)

# 3. load modern occurrence records (latitude and longitude) for your species
species.records <- read.csv("your file directory") #insert your own file directory here
#load modern occurrence records for your species
# columns we used are climate period, latitude, longitude
plot(species.records$longitude, species.records$latitude) #visualize your occurrence records

# spatially thin records within 20km of each other
species.records$species <- "wildcat" # add a column for the species name
species_thinned <- species.records %>% # select relevant columns for thinning
 dplyr::select(latitude,
 longitude,
 species,
 climate.period
 ) %>%
 as.data.frame()

species_thin <- thin(loc.data = species_thinned, # spatially thin records within 4km of each other (you can change this based on your species)
 lat.col = 'latitude',
 long.col = 'longitude',
 spec.col = 'species',
 thin.par = 4, #4km
 reps = 100,
 write.files = FALSE,
 locs.thinned.list.return = TRUE)

sp_thinned <- merge(species_thinned,
 data.frame(Longitude = species_thin[[which.max(sapply(species_thin, nrow))]]$longitude,
 Latitude = species_thin[[which.max(sapply(species_thin, nrow))]]$latitude),
 by = c('longitude','latitude'))[, c('species', 'longitude', 'latitude','climate.period')] #merge the original and thinned datasets

dat_p_thin <- sp_thinned %>% ## add a column to the data set for the occurrence records and specify that these are presence records (= 1)
 mutate(species = 1) %>%
 dplyr::select(species,
 climate.period,
 longitude = Longitude,
 latitude = Latitude) %>%
 as.data.frame()

# 4. Generate background records
dat_current <- dat_p_thin %>% # select relevant columns
 st_as_sf(coords = c("longitude", "latitude"),
 crs = crs(climate)) %>%
 as_Spatial()
presence_buffer <- raster::buffer(dat_current, width = 200000, dissolve = TRUE) # calculate the buffer, dissolving overlapping polygon boundaries
bb_buf <- raster::extent(x = c(min_lon = -10, max_lon = 2, min_lat = 49, max_lat = 62)) # create extent of bounding box as extent of the UK
climate_crop <- raster::crop(x = climate, y = bb_buf) # crop predictor variables to match the study extent
climate_mask <- raster::mask(x = climate_crop, mask = presence_buffer) # mask the predictor variables
dat_current_df <- dat_current %>% # generate a data frame of the presence records that's projection was converted earlier
 as.data.frame() %>%
 dplyr::select(species, Longitude = coords.x1, Latitude = coords.x2)
plot(climate_mask[[1]], main = names(climate_mask)[1])
points(dat_current_df$Longitude, dat_current_df$Latitude, col = "red", pch = 19)
bg_pts <- randomPoints(climate_mask,
 n = 10000 ) %>%
 as.data.frame() %>%
 dplyr::select(Longitude = x, Latitude = y) # sample a selected number of random points from inside the background extent depending on your sample size. Maxents default is 10,000
# convert to a data frame and select the relevant columns
bg_pts <- bg_pts %>%
 add_column(species = 0) %>%
 dplyr::select(species, Longitude, Latitude) # add a column for the background locations and specify that these are background records (=0)
plot(climate_mask[[1]]) # visualise the actual occurrences and the random background occurrences
points(bg_pts$Longitude,
 bg_pts$Latitude,
 pch = ".") # plot the background records
points(dat_behrmann, col = "red", pch = ".", cex = 5)
points(dat_current_df$Longitude,
 dat_current_df$Latitude,
 col = "red",
 cex = 2,
 pch = ".")

pb <- bind_rows(dat_current_df,
 bg_pts) # create a combined data frame with the presence records and background points
# make sure that the columns of the data frames are in the same order
pb_data <- st_as_sf(x = pb,
 coords = c("longitude", "latitude"),
 crs = crs(climate)) # convert the data frame to an sf object and assign the projection to the predictor variables
table(pb_data$species) # check the number of presence and background records

pb_climate <- raster::extract(x = climate, # extract raster values for the species point
 y = pb_data,
 df = TRUE)

pb_climate <- pb_climate[, 2:ncol(pb_climate)] # the extract function creates an ID column that should be excluded for the modelling
pb <- pb_data$species # create a vector of 1 (for presence) and 0 (for background)


pb_data %>% extract(geometry, c('Longitude', 'Latitude'), '\\((.*), (.*)\\)') #split the geometry column into Longitude and Latitude, before detailing the coordinate reference system (crs)

pa_data <- sf::st_as_sf(pb_data, coords = c("Longitude", "Latitude"), crs = 54009) #this crs is the Mollweide equal area projection which is recommended for the Northern Hemisphere

# 5. block cross-validations


# 5 fold spatial blocking by rows and columns with systematic assignment
sb <- cv_spatial(x = pa_data, # presence-background data
 column = "species", # the response column (binary or multi-class)
 k = 5, # number of folds
 size = 50000, # size of the blocks in metres
 selection = "systematic", # random blocks-to-fold
 iteration = 50, # find evenly dispersed folds
 biomod2 = FALSE) # create folds for biomod2


# create empty vectors for evaluations statistics
AUCs <- vector() # area under the curve
CBIs <- vector() #continuous boyce index
OR10s <- vector() # 10% omission rate
ORMTPs <- vector() # maximum training presence
MSSs <- vector() # maximum sensitivity and specificity test

# 6. create species distribution model

folds <- sb$folds # extract folds in buffering object created previously using the 5-fold spatial blocking
for(k in 1:length(folds)){
 trainSet <- unlist(folds[[k]][1]) # extract the training set indices
 testSet <- unlist(folds[[k]][2]) # extract the testing set indices
 # fitting a maxent model using linear, quadratic and product features. The feature classes as well as regularisation multipliers should be chosen based on your study objectives
 mx <- maxnet(pb[trainSet],
 pb_climate[trainSet, ],
 maxnet.formula(pb[trainSet],
 pb_climate[trainSet, ],
 classes = "lqp"))
 testTable <- pa_data[testSet, ] # a table for testing predictions and reference data
 testTable$pred <- predict(mx,
 pb_climate[testSet, ],
 type = "cloglog") # predict the test set
 # evaluation statistics preparation
 # calculate area under the curve (AUC) of the receiver operating curve using the precrec package
 precrec_obj <- evalmod(scores = testTable$pred,
 labels = testTable$species)
 auc <- auc(precrec_obj)[1, 4]
 # calculate the continuous boyce index (CBI) using the ecospat package. The Boyce index only requires presences and measures how much model predictions differ from random distribution of the observed presences across the prediction gradients
 prediction <- predict(mx,
 pb_climate,
 type = 'cloglog')
 cbi <- ecospat.boyce(fit = prediction,
 obs = prediction[pb == 1],
 nclass = 0,
 window.w = "default",
 res = 100,
 PEplot = FALSE)
 # model overfitting and thresholds
 # ORMin or ORMTP = calculates the threshold-dependent metric that indicates the proportion of test localities with suitability of values lower than that associated with the lowest-ranking training locality.
 ORMin <- min(prediction[pb == 1])
 # OR10 = calculates the threshold-dependent metric that indicates the proportion of test localities with suitability values lower than that excluding the 10% of training localities with the lowest predicted suitability
 OR10 <- sort(prediction[pb == 1])[ceiling(length(prediction[pb == 1]) / 10)]
 # MSS = calculates the the threshold at which the sum of the sensitivity (true positive rate) and specificity (true negative rate) is highest
 # specify to run the evaluate() function from dismo due to namespace conflicts
 eval <- dismo::evaluate(p = prediction[pb == 1],
 a = prediction[pb == 0])
 MSS <- eval@t[which.max(eval@TPR + eval@TNR)]
 # from the iterations
 AUCs[k] <- as.numeric(auc)
 CBIs[k] <- as.numeric(cbi$cor)
 ORMTPs[k] <- as.numeric(ORMin)
 OR10s[k] <- as.numeric(OR10)
 MSSs[k] <- as.numeric(MSS)
 # create tibble of the evaluation statistics and thresholds from the block-cross validation
 evaluation_stats <- tibble(
 AUC_mean = round(mean(AUCs), 3),
 AUC_sd = round(sd(AUCs), 3),
 CBI_mean = round(mean(CBIs), 3),
 CBI_sd = round(sd(CBIs), 3),
 ORMTP_mean = round(mean(ORMTPs), 3),
 ORMTP_sd = round(sd(ORMTPs), 3),
 OR10_mean = round(mean(OR10s), 3),
 OR10_sd = round(sd(OR10s), 3),
 MSS_mean = round(mean(MSSs), 3),
 MSS_sd = round(sd(MSSs), 3),
 )
 # fitting a maxent model with the full dataset using linear, quadratic and product features
 me <- maxnet(
 p = pa_data$species,
 data = pb_climate,
 f = maxnet.formula(p = pa_data$species,
 data = pb_climate,
 classes = 'lqp')
 )
 # generate the raster for the maxent model
 final_suitability <- predict(object = climate_crop,
 model = me,
 type = "cloglog")
}


plot(final_suitability) # plot the species distribution model as a continuous map

plot(me, type = "cloglog") # plot the response curves

evaluation_stats # load the model performance evaluation metrics

# 7. Run jackknife test

library(**maxnet**)

library(**precrec**)

species_vec <- as.vector(pa_data$species)

# get variable names

variables <- colnames(pb_climate)

# prepare the variable storage

only_var_auc <- numeric(length(variables))

without_var_auc <- numeric(length(variables))

full_auc <- NA

# run the full model

full_pred <- **predict**(me, pb_climate, type = "cloglog")

full_auc <- **auc**(evalmod(scores = full_pred, labels = species_vec))[1, 4]

# loop for jackknife test through the variable

for (i in seq_along(variables)) {

var <- variables[i]

# model with only one variable

one_var_df <- pb_climate[, var, drop = FALSE]

one_var_df$dummy <- rnorm(nrow(one_var_df)) # Dummy var included for model internals

# train the model

mx_only <- **maxnet**(

species_vec,

one_var_df,

maxnet.formula(species_vec, one_var_df, classes = "lqp")

)

pred_only_df <- one_var_df

pred_only <- **predict**(mx_only, pred_only_df, type = "cloglog")

only_var_auc[i] <- auc(evalmod(scores = pred_only, labels = species_vec))[1, 4]

# create the model without the variable

vars_minus <- **setdiff**(variables, var)

minus_var_df <- pb_climate[, vars_minus, drop = FALSE]

mx_omit <- **maxnet**(

species_vec,

minus_var_df,

maxnet.formula(species_vec, minus_var_df, classes = "lqp")

)

pred_omit <- **predict**(mx_omit, minus_var_df, type = "cloglog")

without_var_auc[i] <- auc(evalmod(scores = pred_omit, labels = species_vec))[1, 4]

}

# combine results

jackknife_results <- **data.frame**(

Variable = variables,

OnlyVarAUC = round(only_var_auc, 3),

WithoutVarAUC = round(without_var_auc, 3),

FullModelAUC = round(full_auc, 3)

)

# print the jackkife results

**print**(jackknife_results)

**Modern historic**

# 1. install and load required packages

install.packages("pacman")

library(pacman)
pacman::p_load(maxnet, rlang, blockCV, dismo, ecospat, precrec, raster,
 sf,tidyverse,rgbif,sp,spThin,maptools,rgeos, rgdal,usdm)

# maxnet is an R package that creates habitat suitability models using Maxent
# rlang is an R package that provides tools for working with core R features and the tidyverse
# blockcv is an R package that creates separate folds for training and testing data for cross-validation
# dismo is an R package that supports habitat suitability models
# ecospat is an R package that supports spatial ecology analysis
# precrec is an R package to calculate ROC curves
# raster is an R package for using rasters
# sf is an R package to implement simple features and encode spatial vector data
# tidyverse is a collection of R packages for data manipulation, exploration and visualization
# rgbif is an R package that allows searching and retrieving data from GBIF
# sp is an R package that provides tools for handling spatial data
# spthin is an R package for spatial thinning of species occurrence records for use in ecological niche models
# maptools is an R package for manipulating spatial data
# rgeos is an R package that performs advanced spatial analysis and manipulation
# rgdal is an R package that allows bindings for spatial analysis, projection and transformation operations
# usdm is an R package that provides functions for exploring the impact of uncertainties on the performance of habitat suitability models


# 2. load environmental (predictor) variables

# current climate data
# bioclimate variables downloaded from worldclim at 2.5 arc second (5km) resolution and crs WGS84
bio17 <- raster("your file directory")# you will need to add your own file directory here where you saved the downloaded climate variables for your species
# name the raster layers after the name of the bioclimatic variable used
# for the wildcat in Britain we used bio 17, 18 and 19
bio18 <- raster("your file directory")
bio19 <- raster("your file directory")


climate <- stack(bio17,bio18,bio19) #stack the current climate variables into a raster stack

slope <- raster ("your file directory") #load your elevation/ topographic variable here
# you will need to add your own file directory here
current <- resample(climate, slope) # use resample to match the geographic extents of the climate stack and slope variable
current <- stack(current, slope) #stack the climate and slope variables into one raster stack
vif(current) # test the variables for collinearity using Variance Inflation Factor (VIF)
# any variable with values <3, indicating high multicollinearity and potential bias, should be excluded from the model


# load HFI data (EPSG: 9001), resample to 2.5 arc minutes, and change crs to WGS 84
hfi <- raster("your file directory")

# stack HFI with climate variables
current <- resample(current, hfi)
climate <- stack(current, hfi)
plot(climate)


# change the names of the raster layers
current_names <- names(climate)
current_names[1] <- "bio17"
current_names[2] <- "bio18"
current_names[3] <- "bio19"
current_names[4] <- "elevation"
current_names[5] <- "human_impact"


# Set the new names to the raster stack
names(climate) <- current_names
plot(climate)


# 3. load occurrence records (latitude and longitude) for your species
# modern records
species.records <- read.csv("your file directory") #insert your own file directory here
#load modern occurrence records for your species
# columns we used are climate period, latitude, longitude
plot(species.records$longitude, species.records$latitude) #visualize your occurrence records

# spatially thin records within 20km of each other
species.records$species <- "wildcat" # add a column for the species name
species_thinned <- species.records %>% # select relevant columns for thinning
 dplyr::select(latitude,
 longitude,
 species,
 climate.period
 ) %>%
 as.data.frame()

species_thin <- thin(loc.data = species_thinned, # spatially thin records within 4km of each other (you can change this based on your species)
 lat.col = 'latitude',
 long.col = 'longitude',
 spec.col = 'species',
 thin.par = 4, #4km
 reps = 100,
 write.files = FALSE,
 locs.thinned.list.return = TRUE)

sp_thinned <- merge(species_thinned,
 data.frame(Longitude = species_thin[[which.max(sapply(species_thin, nrow))]]$longitude,
 Latitude = species_thin[[which.max(sapply(species_thin, nrow))]]$latitude),
 by = c('longitude','latitude'))[, c('species', 'longitude', 'latitude','climate.period')] #merge the original and thinned datasets

dat_p_thin <- sp_thinned %>% ## add a column to the data set for the occurrence records and specify that these are presence records (= 1)
 mutate(species = 1) %>%
 dplyr::select(species,
 climate.period,
 longitude = Longitude,
 latitude = Latitude) %>%
 as.data.frame()


# load historic records
historic.records <- read.csv("your file directory")


plot(historic.records$Longitude, historic.records$Latitude)

# Spatial bias exists in where records were made, but it doesn't necessarily mean that that is the best place for the species in terms of environment. Therefore, we can thin records to maintain a suitable amount of spatial representation without clumping, we select the relevant columns needed for spatial thinning
historic.records$species <- "wildcat"

historic_thinned <- historic.records %>%
 dplyr::select(Latitude,
 Longitude,
 species,
 ) %>%
 as.data.frame()
str(historic_thinned)


# thin the historic records
historic_thin <- thin(loc.data = historic_thinned,
 lat.col = 'Latitude',
 long.col = 'Longitude',
 spec.col = 'species',
 thin.par = 4,
 reps = 100,
 write.files = FALSE,
 locs.thinned.list.return = TRUE)

# Find the maximum presence records in the resulting list
which.max(sapply(historic_thin, nrow))
historic_thin[1]


sp_thinned_h <- merge(historic_thinned,
 data.frame(Longitude = historic_thin[[which.max(sapply(historic_thin, nrow))]]$Longitude,
 Latitude = historic_thin[[which.max(sapply(historic_thin, nrow))]]$Latitude),
 by = c('Longitude','Latitude'))[, c('species', 'Longitude', 'Latitude')]

dat_p_thin_h <- sp_thinned_h %>%
 mutate(species = 1) %>%
 dplyr::select(species,
 Longitude = Longitude,
 Latitude = Latitude) %>%
 as.data.frame()


# 4. Generate background records
species_data <- data_p_thin %>% # select relevant columns
 st_as_sf(coords = c("Longitude", "Latitude"),
 crs = crs(climate)) %>%
 as_Spatial()


presence_buffer <- raster::buffer(species_data, width = 200000, dissolve = TRUE) # calculate the buffer, dissolving overlapping polygon boundaries
bb_buf <- raster::extent(x = c(min_lon = -10, max_lon = 2, min_lat = 49, max_lat = 62)) # create extent of bounding box as extent of the UK
climate_crop <- raster::crop(x = climate, y = bb_buf) #crop predictor variables to match the study extent
climate_mask <- raster::mask(x = climate_crop, mask = presence_buffer) #mask the predictor variables

species_df <- species_data %>% #check your work
 as.data.frame() %>%
 dplyr::select(species, Longitude = coords.x1, Latitude = coords.x2)
plot(climate_mask[[1]], main = names(climate_mask)[1])
points(species_df$Longitude, species_df$Latitude, col = "red", pch = 19)

bg_pts <- randomPoints(climate_mask,
 n = 10000) %>%
 as.data.frame() %>%
 dplyr::select(longitude = x, latitude = y) # sample a selected number of random points from inside the background extent depending on your sample size. Maxents default is 10,000
# convert to a data frame and select the relevant columns
bg_pts <- bg_pts %>%
 add_column(species = 0) %>%
 dplyr::select(species, longitude, latitude) # add a column for the background locations and specify that these are background records (=0)

plot(climate_mask[[1]]) # visualise the actual occurrences and the random background occurrences

pbc <- bind_rows(species_df,
 bg_pts) # create a combined data frame with the presence records and background points
# make sure that the columns of the data frames are in the same order
pb_data_c <- st_as_sf(x = pbc,
 coords = c("longitude", "latitude"),
 crs = crs(climate)) # convert the data frame to an sf object and assign the projection to the predictor variables

table(pb_data_c$species) # check the number of presence and background records
pb_climate_c <- raster::extract(x = climate,
 y = pb_data_c,
 df = TRUE) # extract raster values for presence and background records

pb_climate_c <- pb_climate_c[, 2:ncol(pb_climate_c)] # the extract function creates an ID column that should be excluded for the modelling

pb_c <- pb_data_c$species # create a vector of 1 (for presence) and 0 (for background)


# generate background records for historic records
species_data_h <- historic %>%
 st_as_sf(coords = c("Longitude", "Latitude"),
 crs = crs(climate_h)) %>%
 as_Spatial()

presence_buffer_h <- raster::buffer(species_data_h, width = 200000, dissolve = TRUE)
bb_buf_h <- raster::extent(x = c(min_lon = -10, max_lon = 2, min_lat = 49, max_lat = 62))
climate_crop_h <- raster::crop(x = climate_h, y = bb_buf_h)
climate_mask_h <- raster::mask(x = climate_crop_h, mask = presence_buffer_h)

species_df_h <- species_data_h %>%
 as.data.frame() %>%
 dplyr::select(species, Longitude = coords.x1, Latitude = coords.x2)
plot(climate_mask_h[[1]], main = names(climate_mask_h)[1])
points(species_df_h$Longitude, species_df_h$Latitude, col = "red", pch = 19)

bg_pts_h <- randomPoints(climate_mask_h,
 n = 10000) %>%
 as.data.frame() %>%
 dplyr::select(longitude = x, latitude = y)

bg_pts_h <- bg_pts_h %>%
 add_column(species = 0) %>%
 dplyr::select(species, longitude, latitude)

pb_h <- bind_rows(species_df_h,
 bg_pts_h)
pb_data_h <- st_as_sf(x = pb_h,
 coords = c("longitude", "latitude"),
 crs = crs(climate))
table(pb_data_h$species)


pb_climate_h <- raster::extract(x = climate_h,
 y = pb_data_h,
 df = TRUE)

pb_climate_h <- pb_climate_h[, 2:ncol(pb_climate_h)]
pb_h <- pb_data_h$species


pb_data <- rbind(pb_data_c, pb_data_h) #combine the modern and historic datasets

pb_climate <- bind_rows(pb_climate_c, pb_climate_h) #combine the climate values datasets
pb_climate <- replace(pb_climate, is.na(pb_climate), 0)

pb <- c(pb_c, pb_h) #combine the vectors

pb_data %>% extract(geometry, c('Longitude', 'Latitude'), '\\((.*), (.*)\\)') #split the geometry column into Longitude and Latitude, before detailing the coordinate reference system (crs)
pa_data <- sf::st_as_sf(pb_data, coords = c("Longitude", "Latitude"), crs = 54009) #this crs is the Mollweide equal area projection which is recommended for the Northern Hemisphere

# 5. Block cross-validation
# 5 fold spatial blocking by rows and columns with systematic assignment
sb <- cv_spatial(x = pa_data, # presence-background data
 column = "species", # the response column (binary or multi-class)
 k = 5, # number of folds
 size = 50000, # size of the blocks in metres
 selection = "systematic", # random blocks-to-fold
 iteration = 50, # find evenly dispersed folds
 biomod2 = FALSE) # create folds for biomod2


# create empty vectors for evaluations statistics
AUCs <- vector() # area under the curve
CBIs <- vector() #continuous boyce index
OR10s <- vector() # 10% omission rate
ORMTPs <- vector() # maximum training presence
MSSs <- vector() # maximum sensitivity and specificity test

# 6. create species distribution model

folds <- sb$folds # extract folds in buffering object created previously using the 5-fold spatial blocking
for(k in 1:length(folds)){
 trainSet <- unlist(folds[[k]][1]) # extract the training set indices
 testSet <- unlist(folds[[k]][2]) # extract the testing set indices
 # fitting a maxent model using linear, quadratic and product features. The feature classes as well as regularisation multipliers should be chosen based on your study objectives
 mx <- maxnet(pb[trainSet],
 pb_climate[trainSet, ],
 maxnet.formula(pb[trainSet],
 pb_climate[trainSet, ],
 classes = "lqp"))
 testTable <- pa_data[testSet, ] # a table for testing predictions and reference data
 testTable$pred <- predict(mx,
 pb_climate[testSet, ],
 type = "cloglog") # predict the test set
 # evaluation statistics preparation
 # calculate area under the curve (AUC) of the receiver operating curve using the precrec package
 precrec_obj <- evalmod(scores = testTable$pred,
 labels = testTable$species)
 auc <- auc(precrec_obj)[1, 4]
 # calculate the continuous boyce index (CBI) using the ecospat package. The Boyce index only requires presences and measures how much model predictions differ from random distribution of the observed presences across the prediction gradients
 prediction <- predict(mx,
 pb_climate,
 type = 'cloglog')
 cbi <- ecospat.boyce(fit = prediction,
 obs = prediction[pb == 1],
 nclass = 0,
 window.w = "default",
 res = 100,
 PEplot = FALSE)
 # model overfitting and thresholds
 # ORMin or ORMTP = calculates the threshold-dependent metric that indicates the proportion of test localities with suitability of values lower than that associated with the lowest-ranking training locality.
 ORMin <- min(prediction[pb == 1])
 # OR10 = calculates the threshold-dependent metric that indicates the proportion of test localities with suitability values lower than that excluding the 10% of training localities with the lowest predicted suitability
 OR10 <- sort(prediction[pb == 1])[ceiling(length(prediction[pb == 1]) / 10)]
 # MSS = calculates the the threshold at which the sum of the sensitivity (true positive rate) and specificity (true negative rate) is highest
 # specify to run the evaluate() function from dismo due to namespace conflicts
 eval <- dismo::evaluate(p = prediction[pb == 1],
 a = prediction[pb == 0])
 MSS <- eval@t[which.max(eval@TPR + eval@TNR)]
 # from the iterations
 AUCs[k] <- as.numeric(auc)
 CBIs[k] <- as.numeric(cbi$cor)
 ORMTPs[k] <- as.numeric(ORMin)
 OR10s[k] <- as.numeric(OR10)
 MSSs[k] <- as.numeric(MSS)
 # create tibble of the evaluation statistics and thresholds from the block-cross validation
 evaluation_stats <- tibble(
 AUC_mean = round(mean(AUCs), 3),
 AUC_sd = round(sd(AUCs), 3),
 CBI_mean = round(mean(CBIs), 3),
 CBI_sd = round(sd(CBIs), 3),
 ORMTP_mean = round(mean(ORMTPs), 3),
 ORMTP_sd = round(sd(ORMTPs), 3),
 OR10_mean = round(mean(OR10s), 3),
 OR10_sd = round(sd(OR10s), 3),
 MSS_mean = round(mean(MSSs), 3),
 MSS_sd = round(sd(MSSs), 3),
 )
 # fitting a maxent model with the full dataset using linear, quadratic and product features
 me <- maxnet(
 p = pa_data$species,
 data = pb_climate,
 f = maxnet.formula(p = pa_data$species,
 data = pb_climate,
 classes = 'lqp')
 )
 # generate the raster for the maxent model
 final_suitability <- predict(object = climate_crop,
 model = me,
 type = "cloglog")
}


plot(final_suitability) # plot the species distribution model as a continuous map

plot(me, type = "cloglog") # plot the response curves

evaluation_stats # load the model performance evaluation metrics

# 7. Run jackknife test

library(**maxnet**)

library(**precrec**)

species_vec <- as.vector(pa_data$species)

# get variable names

variables <- colnames(pb_climate)

# prepare the variable storage

only_var_auc <- numeric(length(variables))

without_var_auc <- numeric(length(variables))

full_auc <- NA

# run the full model

full_pred <- **predict**(me, pb_climate, type = "cloglog")

full_auc <- **auc**(evalmod(scores = full_pred, labels = species_vec))[1, 4]

# loop for jackknife test through the variable

for (i in seq_along(variables)) {

var <- variables[i]

# model with only one variable

one_var_df <- pb_climate[, var, drop = FALSE]

one_var_df$dummy <- rnorm(nrow(one_var_df)) # Dummy var included for model internals

# train the model

mx_only <- **maxnet**(

species_vec,

one_var_df,

maxnet.formula(species_vec, one_var_df, classes = "lqp")

)

pred_only_df <- one_var_df

pred_only <- **predict**(mx_only, pred_only_df, type = "cloglog")

only_var_auc[i] <- auc(evalmod(scores = pred_only, labels = species_vec))[1, 4]

# create the model without the variable

vars_minus <- **setdiff**(variables, var)

minus_var_df <- pb_climate[, vars_minus, drop = FALSE]

mx_omit <- **maxnet**(

species_vec,

minus_var_df,

maxnet.formula(species_vec, minus_var_df, classes = "lqp")

)

pred_omit <- **predict**(mx_omit, minus_var_df, type = "cloglog")

without_var_auc[i] <- auc(evalmod(scores = pred_omit, labels = species_vec))[1, 4]

}

# combine results

jackknife_results <- **data.frame**(

Variable = variables,

OnlyVarAUC = round(only_var_auc, 3),

WithoutVarAUC = round(without_var_auc, 3),

FullModelAUC = round(full_auc, 3)

)

# print the jackkife results

**print**(jackknife_results)

**Modern fossil**

# 1. install and load required packages

install.packages("pacman")

library(pacman)
pacman::p_load(maxnet, rlang, blockCV, dismo, ecospat, precrec, raster,
 sf,tidyverse,rgbif,sp,spThin,maptools,rgeos, rgdal,usdm)

# maxnet is an R package that creates habitat suitability models using Maxent
# rlang is an R package that provides tools for working with core R features and the tidyverse
# blockcv is an R package that creates separate folds for training and testing data for cross-validation
# dismo is an R package that supports habitat suitability models
# ecospat is an R package that supports spatial ecology analysis
# precrec is an R package to calculate ROC curves
# raster is an R package for using rasters
# sf is an R package to implement simple features and encode spatial vector data
# tidyverse is a collection of R packages for data manipulation, exploration and visualization
# rgbif is an R package that allows searching and retrieving data from GBIF
# sp is an R package that provides tools for handling spatial data
# spthin is an R package for spatial thinning of species occurrence records for use in ecological niche models
# maptools is an R package for manipulating spatial data
# rgeos is an R package that performs advanced spatial analysis and manipulation
# rgdal is an R package that allows bindings for spatial analysis, projection and transformation operations
# usdm is an R package that provides functions for exploring the impact of uncertainties on the performance of habitat suitability models


# 2. load environmental (predictor) variables

# current climate data
# bioclimate variables downloaded from worldclim at 2.5 arc second (5km) resolution and crs WGS84
bio17 <- raster("your file directory")# you will need to add your own file directory here where you saved the downloaded climate variables for your species
# name the raster layers after the name of the bioclimatic variable used
# for the wildcat in Britain we used bio 17, 18 and 19
bio18 <- raster("your file directory")
bio19 <- raster("your file directory")


climate <- stack(bio17,bio18,bio19) #stack the current climate variables into a raster stack

slope <- raster ("your file directory") #load your elevation/ topographic variable here
# you will need to add your own file directory here
current <- resample(climate, slope) # use resample to match the geographic extents of the climate stack and slope variable
current <- stack(current, slope) #stack the climate and slope variables into one raster stack
vif(current) # test the variables for collinearity using Variance Inflation Factor (VIF)
# any variable with values <3, indicating high multicollinearity and potential bias, should be excluded from the model


# load HFI data (EPSG: 9001), resample to 2.5 arc minutes, and change crs to WGS 84
hfi <- raster("your file directory")

# stack HFI with climate variables
current <- resample(current, hfi)
climate <- stack(current, hfi)
plot(climate)


# change the names of the raster layers
current_names <- names(climate)
current_names[1] <- "bio17"
current_names[2] <- "bio18"
current_names[3] <- "bio19"
current_names[4] <- "elevation"
current_names[5] <- "human_impact"

# fossil climate data
#late Holocene
#palaeoclimate variables downloaded from palaeoclim at 2.5 arc second (5km) resolution and crs WGS84
LH_bio17 <- raster("your file directory")
LH_bio18 <- raster("your file directory")
LH_bio19 <- raster("your file directory")


climate_m <- stack(LH_bio17,LH_bio18,LH_bio19) #stack the current climate variables into a raster stack

plot(climate_m)

meg_env <- resample(climate_m, slope) #resample the slope and climate variables to the same extent
mg_env <- stack(meg_env, slope) #stack the climate and slope variables
plot(mg_env)


hfi_m <- raster("your file here") #load the fossil hfi data (values are changed to 0 indicating no human impact)

current <- resample(mg_env, hfi_m) #resample the hfi layer to 2.5 arc minute resolution
mg_env <- stack(current, hfi_m) #stack the hfi, climate and slope data
plot(mg_env)

#change stack layer names
current_names <- names(mg_env)
current_names[1] <- "bio17"
current_names[2] <- "bio18"
current_names[3] <- "bio19"
current_names[4] <- "elevation"
current_names[5] <- "human_impact"


names(mg_env) <- current_names
m_env <- mg_env
plot(m_env)


# mid Holocene
#load climate variables
ng_bio17 <- raster("your file directory here")
ng_bio18 <- raster("your file directory here")
ng_bio19 <- raster("your file directory here")

climate_n <- stack(ng_bio17,ng_bio18,ng_bio19)
plot(climate_n)
n_env <- resample(climate_n, slope) # resample and stack
ng_env <- stack(n_env, slope)


hfi_n <- raster("your file directory here") #load the fossil hfi data (values are changed to 0 indicating no human impact)

current <- resample(ng_env, hfi_n) #resample and stack the hfi variable with the climate and slope variables
ng_env <- stack(current, hfi_n)
plot(ng_env)

#change stack layer names
current_names <- names(ng_env)
current_names[1] <- "bio17"
current_names[2] <- "bio18"
current_names[3] <- "bio19"
current_names[4] <- "elevation"
current_names[5] <- "human_impact"
names(ng_env) <- current_names
plot(ng_env)

#early Holocene
#load climate variables
g_bio17 <- raster("your file directory here")
g_bio18 <- raster("your file directory here")
g_bio19 <- raster("your file directory here")

climate_g <- stack(g_bio17,g_bio18,g_bio19)
plot(climate_g)


g_env <- resample #resample and stack
gl_env <- stack(g_env, slope)
plot(gl_env)


hfi_g <- raster("your directory here") #load the fossil hfi data (values are changed to 0 indicating no human impact)

current <- resample(gl_env, hfi_g) #resample and stack hfi with slope and climate variables
gl_env <- stack(current, hfi_g)
plot(gl_env)

#change stack layer names
current_names <- names(gl_env)
current_names[1] <- "bio17"
current_names[2] <- "bio18"
current_names[3] <- "bio19"
current_names[4] <- "elevation"
current_names[5] <- "human_impact"
names(gl_env) <- current_names
plot(gl_env)

# 3. load occurrence records (latitude and longitude) for your species
# modern records
species.records <- read.csv("your file directory") #insert your own file directory here
#load modern occurrence records for your species
# columns we used are climate period, latitude, longitude
plot(species.records$longitude, species.records$latitude) #visualize your occurrence records

# spatially thin records within 20km of each other
species.records$species <- "wildcat" # add a column for the species name
species_thinned <- species.records %>% # select relevant columns for thinning
 dplyr::select(latitude,
 longitude,
 species,
 climate.period
 ) %>%
 as.data.frame()

species_thin <- thin(loc.data = species_thinned, # spatially thin records within 4km of each other (you can change this based on your species)
 lat.col = 'latitude',
 long.col = 'longitude',
 spec.col = 'species',
 thin.par = 4, #4km
 reps = 100,
 write.files = FALSE,
 locs.thinned.list.return = TRUE)

sp_thinned <- merge(species_thinned,
 data.frame(Longitude = species_thin[[which.max(sapply(species_thin, nrow))]]$longitude,
 Latitude = species_thin[[which.max(sapply(species_thin, nrow))]]$latitude),
 by = c('longitude','latitude'))[, c('species', 'longitude', 'latitude','climate.period')] #merge the original and thinned datasets

dat_p_thin <- sp_thinned %>% ## add a column to the data set for the occurrence records and specify that these are presence records (= 1)
 mutate(species = 1) %>%
 dplyr::select(species,
 climate.period,
 longitude = Longitude,
 latitude = Latitude) %>%
 as.data.frame()


# load historic records
historic.records <- read.csv("your file directory")


plot(historic.records$Longitude, historic.records$Latitude)

# Spatial bias exists in where records were made, but it doesn't necessarily mean that that is the best place for the species in terms of environment. Therefore, we can thin records to maintain a suitable amount of spatial representation without clumping, we select the relevant columns needed for spatial thinning
historic.records$species <- "wildcat"

historic_thinned <- historic.records %>%
 dplyr::select(Latitude,
 Longitude,
 species,
 ) %>%
 as.data.frame()
str(historic_thinned)


# thin the historic records
historic_thin <- thin(loc.data = historic_thinned,
 lat.col = 'Latitude',
 long.col = 'Longitude',
 spec.col = 'species',
 thin.par = 4,
 reps = 100,
 write.files = FALSE,
 locs.thinned.list.return = TRUE)

# Find the maximum presence records in the resulting list
which.max(sapply(historic_thin, nrow))
historic_thin[1]


sp_thinned_h <- merge(historic_thinned,
 data.frame(Longitude = historic_thin[[which.max(sapply(historic_thin, nrow))]]$Longitude,
 Latitude = historic_thin[[which.max(sapply(historic_thin, nrow))]]$Latitude),
 by = c('Longitude','Latitude'))[, c('species', 'Longitude', 'Latitude')]

dat_p_thin_h <- sp_thinned_h %>%
 mutate(species = 1) %>%
 dplyr::select(species,
 Longitude = Longitude,
 Latitude = Latitude) %>%
 as.data.frame()


# late Holocene (Meghalayan)
meg_records <- read.csv("your file directory here") #load csv file of records (latitude and longitude)
plot(meg_records$Longitude, meg_records$Latitude)

meg_records$species <- "wildcat" #add species name

meg_thinned <- meg_records %>% #select relevant columns for thinning
 dplyr::select(Latitude,
 Longitude,
 species,
 ) %>%
 as.data.frame()
str(meg_thinned)


meg_thin <- thin(loc.data = meg_thinned, #thin records within 4km of each other
 lat.col = 'Latitude',
 long.col = 'Longitude',
 spec.col = 'species',
 thin.par = 4,
 reps = 100,
 write.files = FALSE,
 locs.thinned.list.return = TRUE)


sp_thinned_m <- merge(meg_thinned,
 data.frame(Longitude = meg_thin[[which.max(sapply(meg_thin, nrow))]]$Longitude,
 Latitude = meg_thin[[which.max(sapply(meg_thin, nrow))]]$Latitude),
 by = c('Longitude','Latitude'))[, c('species', 'Longitude', 'Latitude')]

dat_p_thin_m <- sp_thinned_m %>%
 mutate(species = 1) %>%
 dplyr::select(species,
 Longitude = Longitude,
 Latitude = Latitude) %>%
 as.data.frame() #merge the original and thinned datasets


# mid Holocene (Northgrippian)
north_records <- read.csv("your file directory here") #load csv file
plot(north_records$Longitude, north_records$Latitude)

north_records$species <- "wildcat" #add column for species name

n_thinned <- north_records %>% #select relevant columns for thinning
 dplyr::select(Latitude,
 Longitude,
 species,
 ) %>%
 as.data.frame()
str(n_thinned)


n_thin <- thin(loc.data = n_thinned, #spatially thin records by 4km
 lat.col = 'Latitude',
 long.col = 'Longitude',
 spec.col = 'species',
 thin.par = 4,
 reps = 100,
 write.files = FALSE,
 locs.thinned.list.return = TRUE)


sp_thinned_n <- merge(n_thinned,
 data.frame(Longitude = n_thin[[which.max(sapply(n_thin, nrow))]]$Longitude,
 Latitude = n_thin[[which.max(sapply(n_thin, nrow))]]$Latitude),
 by = c('Longitude','Latitude'))[, c('species', 'Longitude', 'Latitude')]

dat_p_thin_n <- sp_thinned_n %>%
 mutate(species = 1) %>%
 dplyr::select(species,
 Longitude = Longitude,
 Latitude = Latitude) %>%
 as.data.frame() #merge the thinned and original datasets


#early Holocene (Greenlandian)
green_records <- read.csv("your file directory here") #load csv file
plot(green_records$Longitude, green_records$Latitude)


green_records$species <- "wildcat" #add column for species name


g_thinned <- green_records %>% #select relevant columns for thinning
 dplyr::select(Latitude,
 Longitude,
 species,
 ) %>%
 as.data.frame()
str(g_thinned)


g_thin <- thin(loc.data = g_thinned, #spatially thin records by 4km
 lat.col = 'Latitude',
 long.col = 'Longitude',
 spec.col = 'species',
 thin.par = 4,
 reps = 100,
 write.files = FALSE,
 locs.thinned.list.return = TRUE)


sp_thinned_g <- merge(g_thinned,
 data.frame(Longitude = g_thin[[which.max(sapply(g_thin, nrow))]]$Longitude,
 Latitude = g_thin[[which.max(sapply(g_thin, nrow))]]$Latitude),
 by = c('Longitude','Latitude'))[, c('species', 'Longitude', 'Latitude')]

dat_p_thin_g <- sp_thinned_g %>%
 mutate(species = 1) %>%
 dplyr::select(species,
 Longitude = Longitude,
 Latitude = Latitude) %>%
 as.data.frame() #merge the thinned and original datasets

# 4. Generate background records
# we generate background records for each individual time period (modern, historic, late, mid and early Holocene)

# modern records
species_data <- data_p_thin %>% # select relevant columns
 st_as_sf(coords = c("Longitude", "Latitude"),
 crs = crs(climate)) %>%
 as_Spatial()


presence_buffer <- raster::buffer(species_data, width = 200000, dissolve = TRUE) # calculate the buffer, dissolving overlapping polygon boundaries
bb_buf <- raster::extent(x = c(min_lon = -10, max_lon = 2, min_lat = 49, max_lat = 62)) # create extent of bounding box as extent of the UK
climate_crop <- raster::crop(x = climate, y = bb_buf) #crop predictor variables to match the study extent
climate_mask <- raster::mask(x = climate_crop, mask = presence_buffer) #mask the predictor variables

species_df <- species_data %>% #check your work
 as.data.frame() %>%
 dplyr::select(species, Longitude = coords.x1, Latitude = coords.x2)
plot(climate_mask[[1]], main = names(climate_mask)[1])
points(species_df$Longitude, species_df$Latitude, col = "red", pch = 19)

bg_pts <- randomPoints(climate_mask,
 n = 10000) %>%
 as.data.frame() %>%
 dplyr::select(longitude = x, latitude = y) # sample a selected number of random points from inside the background extent depending on your sample size. Maxents default is 10,000
# convert to a data frame and select the relevant columns

bg_pts <- bg_pts %>%
 add_column(species = 0) %>%
 dplyr::select(species, longitude, latitude) # add a column for the background locations and specify that these are background records (=0)

plot(climate_mask[[1]]) # visualise the actual occurrences and the random background occurrences

pbc <- bind_rows(species_df,
 bg_pts) # create a combined data frame with the presence records and background points
# make sure that the columns of the data frames are in the same order

pb_data_c <- st_as_sf(x = pbc,
 coords = c("longitude", "latitude"),
 crs = crs(climate)) # convert the data frame to an sf object and assign the projection to the predictor variables

table(pb_data_c$species) # check the number of presence and background records


pb_climate_c <- raster::extract(x = climate,
 y = pb_data_c,
 df = TRUE) # extract raster values for the species point

pb_climate_c <- pb_climate_c[, 2:ncol(pb_climate_c)] # the extract function creates an ID column that should be excluded for the modelling

pb_c <- pb_data_c$species # create a vector of 1 (for presence) and 0 (for background)


# historic records
species_data_h <- historic %>%
 st_as_sf(coords = c("Longitude", "Latitude"),
 crs = crs(climate_h)) %>%
 as_Spatial()

presence_buffer_h <- raster::buffer(species_data_h, width = 200000, dissolve = TRUE)
bb_buf_h <- raster::extent(x = c(min_lon = -10, max_lon = 2, min_lat = 49, max_lat = 62))
climate_crop_h <- raster::crop(x = climate_h, y = bb_buf_h)
climate_mask_h <- raster::mask(x = climate_crop_h, mask = presence_buffer_h)

species_df_h <- species_data_h %>%
 as.data.frame() %>%
 dplyr::select(species, Longitude = coords.x1, Latitude = coords.x2)
plot(climate_mask_h[[1]], main = names(climate_mask_h)[1])
points(species_df_h$Longitude, species_df_h$Latitude, col = "red", pch = 19)

bg_pts_h <- randomPoints(climate_mask_h,
 n = 10000) %>%
 as.data.frame() %>%
 dplyr::select(longitude = x, latitude = y)

bg_pts_h <- bg_pts_h %>%
 add_column(species = 0) %>%
 dplyr::select(species, longitude, latitude)

pb_h <- bind_rows(species_df_h,
 bg_pts_h)
pb_data_h <- st_as_sf(x = pb_h,
 coords = c("longitude", "latitude"),
 crs = crs(climate))
table(pb_data_h$species)


pb_climate_h <- raster::extract(x = climate_h,
 y = pb_data_h,
 df = TRUE)

pb_climate_h <- pb_climate_h[, 2:ncol(pb_climate_h)]
pb_h <- pb_data_h$species


# late Holocene (Meghalayan)
species_data_m <- meghalayan %>%
 st_as_sf(coords = c("Longitude", "Latitude"),
 crs = crs(m_env)) %>%
 as_Spatial()

presence_buffer_m <- raster::buffer(species_data_m, width = 200000, dissolve = TRUE)
# calculate the buffer size around the points
bb_buf_m <- raster::extent(x = c(min_lon = -10, max_lon = 2, min_lat = 49, max_lat = 62)) # create a bounding box with the extent set as the UK
climate_crop_m <- raster::crop(x = m_env, y = bb_buf_m) #crop the environmental predictor variables to match the study extent
climate_mask_m <- raster::mask(x = climate_crop_m, mask = presence_buffer_m)

species_df_m <- species_data_m %>% # plot the presence records and climate buffer
 as.data.frame() %>%
 dplyr::select(species, Longitude = coords.x1, Latitude = coords.x2)
plot(climate_mask_m[[1]], main = names(climate_mask_m)[1])
points(species_df_m$Longitude, species_df_m$Latitude, col = "red", pch = 19)


bg_pts_m <- randomPoints(climate_mask_m, # generate 10,000 random points from the background extent
 n = 10000) %>%
 as.data.frame() %>%
 dplyr::select(longitude = x, latitude = y)

bg_pts_m <- bg_pts_m %>% # add a column for the backgorund points and give a value of 0 to specify they are absence records
 add_column(species = 0) %>%
 dplyr::select(species, longitude, latitude)


pb_m <- bind_rows(species_df_m, # create a single dataframe with the presence and background records
 bg_pts_m)
pb_data_m <- st_as_sf(x = pb_m, # convert the data frame to an sf object
 coords = c("longitude", "latitude"),
 crs = crs(m_env))
table(pb_data_m$species)

pb_climate_m <- raster::extract(x = m_env, # extract the environmental predictor values for the presence and background points
 y = pb_data_m,
 df = TRUE)

pb_climate_m <- pb_climate_m[, 2:ncol(pb_climate_m)] # remove unnecessary columns

pb_m <- pb_data_m$species #create a vector of 1 for presence and 0 for background


# mid Holocene (northgrippian)

species_data_n <- northgrippian %>%
 st_as_sf(coords = c("Longitude", "Latitude"),
 crs = crs(ng_env)) %>%
 as_Spatial()

presence_buffer_n <- raster::buffer(species_data_n, width = 200000, dissolve = TRUE)
bb_buf_n <- raster::extent(x = c(min_lon = -10, max_lon = 2, min_lat = 49, max_lat = 62))
climate_crop_n <- raster::crop(x = ng_env, y = bb_buf_n)
climate_mask_n <- raster::mask(x = climate_crop_n, mask = presence_buffer_n)

species_df_n <- species_data_n %>%
 as.data.frame() %>%
 dplyr::select(species, Longitude = coords.x1, Latitude = coords.x2)
plot(climate_mask_n[[1]], main = names(climate_mask_n)[1])
points(species_df_n$Longitude, species_df_n$Latitude, col = "red", pch = 19)

bg_pts_n <- randomPoints(climate_mask_n, #sample 10,000 background points
 n = 10000) %>%
 as.data.frame() %>%
 dplyr::select(longitude = x, latitude = y)

bg_pts_n <- bg_pts_n %>% #specify these as background points
 add_column(species = 0) %>%
 dplyr::select(species, longitude, latitude)
pb_n <- bind_rows(species_df_n, #combine presence and background points
 bg_pts_n)
pb_data_n <- st_as_sf(x = pb_n, #convert to an sf object
 coords = c("longitude", "latitude"),
 crs = crs(ng_env))
table(pb_data_n$species)

pb_climate_n <- raster::extract(x = ng_env, # extract raster values for each layer
 y = pb_data_n,
 df = TRUE)

pb_climate_n <- pb_climate_n[, 2:ncol(pb_climate_n)] #remove unnecessary columns
pb_n <- pb_data_n$species


# early Holocene (Greenlandian)
species_data_g <- greenlandian %>%
 st_as_sf(coords = c("Longitude", "Latitude"),
 crs = crs(gl_env)) %>%
 as_Spatial()

presence_buffer_g <- raster::buffer(species_data_g, width = 200000, dissolve = TRUE)
bb_buf_g <- raster::extent(x = c(min_lon = -10, max_lon = 2, min_lat = 49, max_lat = 62))
climate_crop_g <- raster::crop(x = gl_env, y = bb_buf_g)
climate_mask_g <- raster::mask(x = climate_crop_g, mask = presence_buffer_g)

species_df_g <- species_data_g %>%
 as.data.frame() %>%
 dplyr::select(species, Longitude = coords.x1, Latitude = coords.x2)
plot(climate_mask_g[[1]], main = names(climate_mask_g)[1])
points(species_df_g$Longitude, species_df_g$Latitude, col = "red", pch = 19)


bg_pts_g <- randomPoints(climate_mask_g, #sample 10,000 background points
 n = 10000) %>%
 as.data.frame() %>%
 dplyr::select(longitude = x, latitude = y)

bg_pts_g <- bg_pts_g %>% #specify these as background points (value =0)
 add_column(species = 0) %>%
 dplyr::select(species, longitude, latitude)

pb_g <- bind_rows(species_df_g, #combine the presence and background points into one data frame
 bg_pts_g)

pb_data_g <- st_as_sf(x = pb_g, #convert into an sf object
 coords = c("longitude", "latitude"),
 crs = crs(gl_env))
table(pb_data_g$species)

pb_climate_g <- raster::extract(x = gl_env, #extract the raster values for each predictor variable
 y = pb_data_g,
 df = TRUE)

pb_climate_g <- pb_climate_g[, 2:ncol(pb_climate_g)] # remove unnecessary columns
pb_g <- pb_data_g$species # create a vector with 1 for presence and 0 for background

pb_data <- rbind(pb_data_c, pb_data_h, pb_data_m, pb_data_n, pb_data_g) #combine all the individual sf objects into one
pb_climate <- bind_rows(pb_climate_c, pb_climate_h, pb_climate_m, pb_climate_n, pb_climate_g) #combine all the extracted raster values into one
pb <- c(pb_c, pb_h, pb_m, pb_n, pb_g) #combine all the vectors into one

pb_data %>% extract(geometry, c('Longitude', 'Latitude'), '\\((.*), (.*)\\)') #split the geometry column into lat and long

pa_data <- sf::st_as_sf(pb_data, coords = c("Longitude", "Latitude"), crs = 54009) #specify the crs
#this crs is the Mollweide equal area projection which is recommended for the Northern Hemisphere


# 5. Block cross-validation
# 5 fold spatial blocking by rows and columns with systematic assignment
sb <- cv_spatial(x = pa_data, # presence-background data
 column = "species", # the response column (binary or multi-class)
 k = 5, # number of folds
 size = 50000, # size of the blocks in metres
 selection = "systematic", # random blocks-to-fold
 iteration = 50, # find evenly dispersed folds
 biomod2 = FALSE) # create folds for biomod2


# create empty vectors for evaluations statistics
AUCs <- vector() # area under the curve
CBIs <- vector() #continuous boyce index
OR10s <- vector() # 10% omission rate
ORMTPs <- vector() # maximum training presence
MSSs <- vector() # maximum sensitivity and specificity test

# 6. create species distribution model

folds <- sb$folds # extract folds in buffering object created previously using the 5-fold spatial blocking
for(k in 1:length(folds)){
 trainSet <- unlist(folds[[k]][1]) # extract the training set indices
 testSet <- unlist(folds[[k]][2]) # extract the testing set indices
 # fitting a maxent model using linear, quadratic and product features. The feature classes as well as regularisation multipliers should be chosen based on your study objectives
 mx <- maxnet(pb[trainSet],
 pb_climate[trainSet, ],
 maxnet.formula(pb[trainSet],
 pb_climate[trainSet, ],
 classes = "lqp"))
 testTable <- pa_data[testSet, ] # a table for testing predictions and reference data
 testTable$pred <- predict(mx,
 pb_climate[testSet, ],
 type = "cloglog") # predict the test set
 # evaluation statistics preparation
 # calculate area under the curve (AUC) of the receiver operating curve using the precrec package
 precrec_obj <- evalmod(scores = testTable$pred,
 labels = testTable$species)
 auc <- auc(precrec_obj)[1, 4]
 # calculate the continuous boyce index (CBI) using the ecospat package. The Boyce index only requires presences and measures how much model predictions differ from random distribution of the observed presences across the prediction gradients
 prediction <- predict(mx,
 pb_climate,
 type = 'cloglog')
 cbi <- ecospat.boyce(fit = prediction,
 obs = prediction[pb == 1],
 nclass = 0,
 window.w = "default",
 res = 100,
 PEplot = FALSE)
 # model overfitting and thresholds
 # ORMin or ORMTP = calculates the threshold-dependent metric that indicates the proportion of test localities with suitability of values lower than that associated with the lowest-ranking training locality.
 ORMin <- min(prediction[pb == 1])
 # OR10 = calculates the threshold-dependent metric that indicates the proportion of test localities with suitability values lower than that excluding the 10% of training localities with the lowest predicted suitability
 OR10 <- sort(prediction[pb == 1])[ceiling(length(prediction[pb == 1]) / 10)]
 # MSS = calculates the the threshold at which the sum of the sensitivity (true positive rate) and specificity (true negative rate) is highest
 # specify to run the evaluate() function from dismo due to namespace conflicts
 eval <- dismo::evaluate(p = prediction[pb == 1],
 a = prediction[pb == 0])
 MSS <- eval@t[which.max(eval@TPR + eval@TNR)]
 # from the iterations
 AUCs[k] <- as.numeric(auc)
 CBIs[k] <- as.numeric(cbi$cor)
 ORMTPs[k] <- as.numeric(ORMin)
 OR10s[k] <- as.numeric(OR10)
 MSSs[k] <- as.numeric(MSS)
 # create tibble of the evaluation statistics and thresholds from the block-cross validation
 evaluation_stats <- tibble(
 AUC_mean = round(mean(AUCs), 3),
 AUC_sd = round(sd(AUCs), 3),
 CBI_mean = round(mean(CBIs), 3),
 CBI_sd = round(sd(CBIs), 3),
 ORMTP_mean = round(mean(ORMTPs), 3),
 ORMTP_sd = round(sd(ORMTPs), 3),
 OR10_mean = round(mean(OR10s), 3),
 OR10_sd = round(sd(OR10s), 3),
 MSS_mean = round(mean(MSSs), 3),
 MSS_sd = round(sd(MSSs), 3),
 )
 # fitting a maxent model with the full dataset using linear, quadratic and product features
 me <- maxnet(
 p = pa_data$species,
 data = pb_climate,
 f = maxnet.formula(p = pa_data$species,
 data = pb_climate,
 classes = 'lqp')
 )
 # generate the raster for the maxent model
 final_suitability <- predict(object = climate_crop,
 model = me,
 type = "cloglog")
}


plot(final_suitability) # plot the species distribution model as a continuous map

plot(me, type = "cloglog") # plot the response curves

evaluation_stats # load the model performance evaluation metrics

# 7. Run jackknife test

library(**maxnet**)

library(**precrec**)

species_vec <- as.vector(pa_data$species)

# get variable names

variables <- colnames(pb_climate)

# prepare the variable storage

only_var_auc <- numeric(length(variables))

without_var_auc <- numeric(length(variables))

full_auc <- NA

# run the full model

full_pred <- **predict**(me, pb_climate, type = "cloglog")

full_auc <- **auc**(evalmod(scores = full_pred, labels = species_vec))[1, 4]

# loop for jackknife test through the variable

for (i in seq_along(variables)) {

var <- variables[i]

# model with only one variable

one_var_df <- pb_climate[, var, drop = FALSE]

one_var_df$dummy <- rnorm(nrow(one_var_df)) # Dummy var included for model internals

# train the model

mx_only <- **maxnet**(

species_vec,

one_var_df,

maxnet.formula(species_vec, one_var_df, classes = "lqp")

)

pred_only_df <- one_var_df

pred_only <- **predict**(mx_only, pred_only_df, type = "cloglog")

only_var_auc[i] <- auc(evalmod(scores = pred_only, labels = species_vec))[1, 4]

# create the model without the variable

vars_minus <- **setdiff**(variables, var)

minus_var_df <- pb_climate[, vars_minus, drop = FALSE]

mx_omit <- **maxnet**(

species_vec,

minus_var_df,

maxnet.formula(species_vec, minus_var_df, classes = "lqp")

)

pred_omit <- **predict**(mx_omit, minus_var_df, type = "cloglog")

without_var_auc[i] <- auc(evalmod(scores = pred_omit, labels = species_vec))[1, 4]

}

# combine results

jackknife_results <- **data.frame**(

Variable = variables,

OnlyVarAUC = round(only_var_auc, 3),

WithoutVarAUC = round(without_var_auc, 3),

FullModelAUC = round(full_auc, 3)

)

# print the jackkife results

**print**(jackknife_results)
